# Construction of an artificial phosphoketolase pathway that efficiently catabolizes multiple carbon sources to acetyl-CoA

**DOI:** 10.1371/journal.pbio.3002285

**Published:** 2023-09-21

**Authors:** Yiqun Yang, Yuwan Liu, Haodong Zhao, Dingyu Liu, Jie Zhang, Jian Cheng, Qiaoyu Yang, Huanyu Chu, Xiaoyun Lu, Mengting Luo, Xiang Sheng, Yi-Heng P. J. Zhang, Huifeng Jiang, Yanhe Ma

**Affiliations:** 1 Tianjin Institute of Industrial Biotechnology, Chinese Academy of Sciences, Tianjin, China; 2 National Center of Technology Innovation for Synthetic Biology, Tianjin, China; 3 Haihe Laboratory of Synthetic Biology, Tianjin, China; 4 School of Life Sciences, University of Science and Technology of China, Hefei, Anhui, China; 5 School of Life Science and Technology, Wuhan Polytechnic University, Wuhan, China; National University of Singapore, SINGAPORE

## Abstract

The canonical glycolysis pathway is responsible for converting glucose into 2 molecules of acetyl-coenzyme A (acetyl-CoA) through a cascade of 11 biochemical reactions. Here, we have designed and constructed an artificial phosphoketolase (APK) pathway, which consists of only 3 types of biochemical reactions. The core enzyme in this pathway is phosphoketolase, while phosphatase and isomerase act as auxiliary enzymes. The APK pathway has the potential to achieve a 100% carbon yield to acetyl-CoA from any monosaccharide by integrating a one-carbon condensation reaction. We tested the APK pathway in vitro, demonstrating that it could efficiently catabolize typical C1-C6 carbohydrates to acetyl-CoA with yields ranging from 83% to 95%. Furthermore, we engineered *Escherichia coli* stain capable of growth utilizing APK pathway when glycerol act as a carbon source. This novel catabolic pathway holds promising route for future biomanufacturing and offering a stoichiometric production platform using multiple carbon sources.

## Abstract 2

The canonical glycolysis pathway converts glucose into two molecules of acetyl-CoA via a cascade of eleven biochemical reactions. This study repurposes phosphoketolase to construct an artificial pathway which comprises only three reactions and could theoretically achieve a 100% carbon yield of acetyl-CoA from any sugar.

## Introduction

Glycolysis, a catabolic pathway comprising 10 cascade biochemical reactions, is responsible for converting glucose to pyruvate, which is further decarboxylated to produce acetyl-CoA. This process serves as an essential energy source and supplier of building blocks for life [1,2]. Over billions of years, natural selection has evolved intricate networks within glycolysis to maintain metabolic and physiological homeostasis [3–5]. While intensive efforts have been devoted to redirecting metabolic homeostasis toward desired compounds with the maximum stoichiometry [6–10], the synthesis of cellular metabolites via glycolysis has been evolved with the optimality principles in nature [11,12]. Partially engineering the central carbon metabolism is insufficient to unravel the complexities of intracellular catabolic networks [13,14]. In this study, we capitalized on the chemical principles to design and implement a de novo pathway called artificial phosphoketolase (APK), which leverages 3 core reactions to convert any ketose into acetyl-CoA.

**Fig 1:**
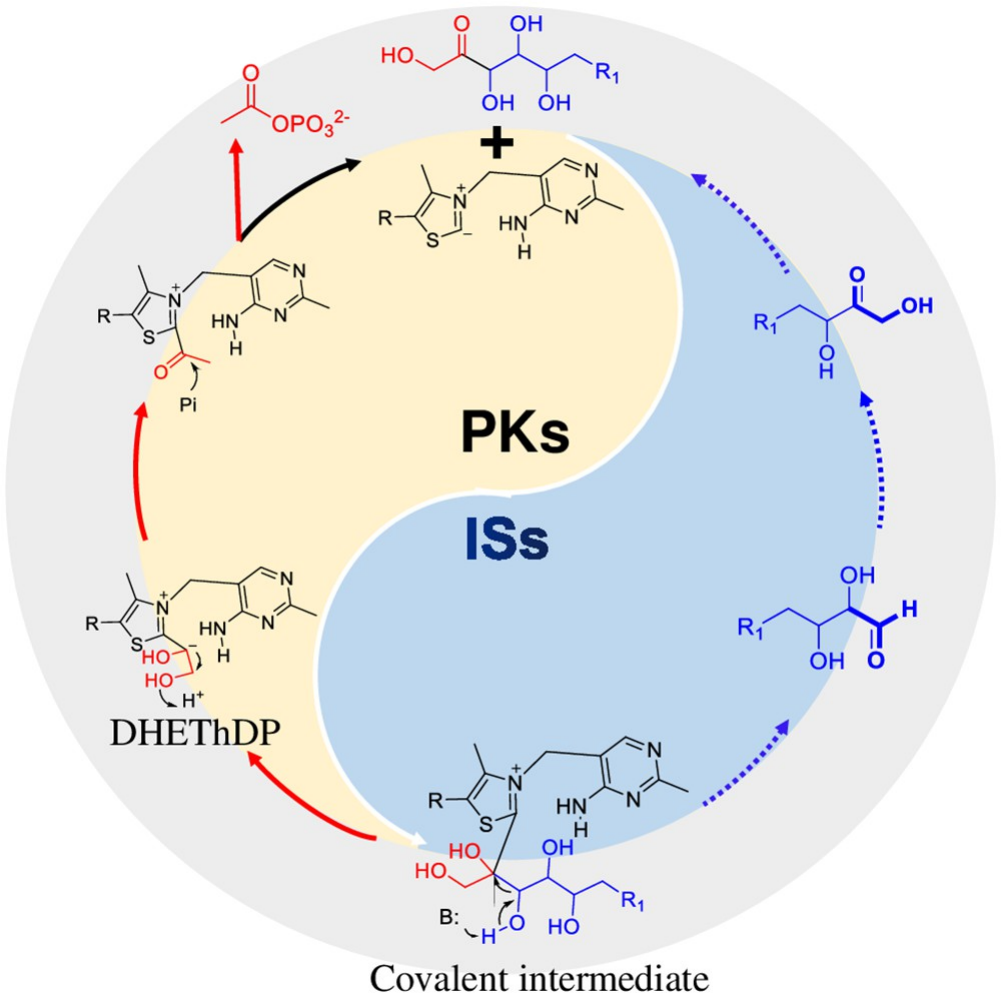
The design of APK pathway. The forming process of AcP from ketoses by PKs is indicated by the red arrows. The blue arrows represent the conversion of aldose molecules to ketose molecules by the action of ISs. AcP, acetyl-phosphate; APK, artificial phosphoketolase; IS, isomerase; PK, phosphoketolase.

Among the key enzymes involved in C–C bond cleavage, thiamin diphosphate (ThDP)-dependent phosphoketolase (PK) plays a significant role [15,16]. PKs have the ability to break the C2-C3 bond of diverse ketoses, generating acetyl-phosphate (AcP) as a product. This AcP can then be further converted to acetyl-CoA by phosphate acetyltransferase (PTA) [17]. According to the catalytic mechanisms of PK, it is possible that ThDP could accept any ketose, forming a covalent intermediate that yields AcP and releases a free aldose (Fig 1) [15]. At the same time, isomerases enable the interconversion between aldoses and ketoses [18,19]. Therefore, we hypothesized that all ketoses could be completely converted to acetyl-CoA through multiple cycles of carbon cleavage by PK and isomerization (Fig 1). Inspired by these natural and hypothetical catalytic processes, we proposed the construction of the APK pathway to facilitate the production of stoichiometric quantities of acetyl-CoA from any sugar.

## Results and discussion

**Fig 2:**
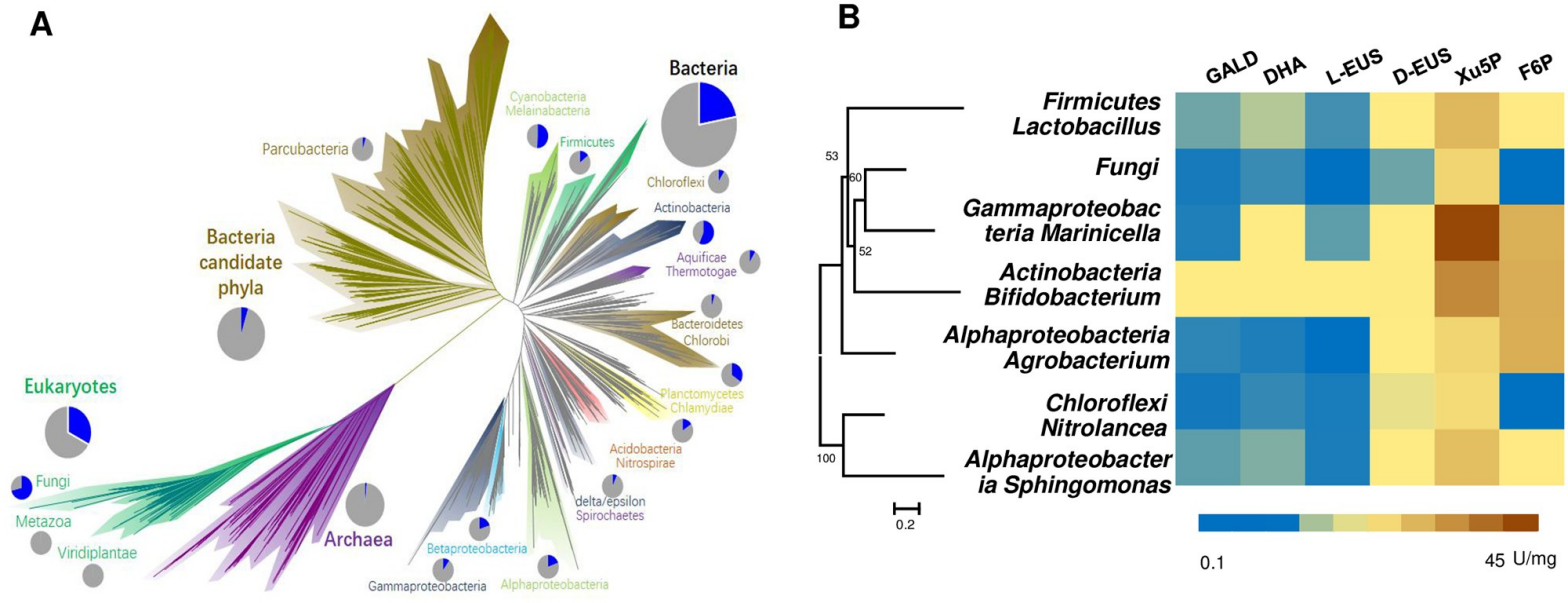
The distribution and new functions of PKs. (**A**) The distribution of PKs across the tree of life, as referenced in Jillian F. Banfield’s study [38]. The blue color in pie chart represents species with PKs present in all genome sequenced species. (**B**) The catalytic activity of PKs from different species on different substrates. The displayed 7 PKs not only exhibited activity on F6P or Xu5P, but also demonstrated the ability to convert short-chain ketoses into AcP. The corresponding table on the right represents the catalytic activity of these 7 candidate PKs on 6 classes of ketose or ketose phosphate. Each color represents to a specific enzyme activity (U/mg). Detailed catalytic activity data are shown in S1 Table. The raw data was listed in S1 Data. AcP, acetyl-phosphate; F6P, fructose-6-phosphate; PK, phosphoketolase; Xu5P, xylulose-5-phosphate.

Indeed, the PK gene has originated very early in nature and is widely distributed among the 3 kingdoms of life, indicating its extensive application in carbon metabolism throughout evolutionary history (Figs 2A and S1). To assess the activity of PK on different sugars, we selected 23 candidate genes from different species based on the phylogenetic tree analysis (S2 Fig). However, due to significant evolutionary distances, only 11 out of 23 PK genes were successfully expressed in *Escherichia coli*. Among these 11 candidates, 7 PKs displayed activity not only on fructose-6-phosphate (F6P) or xylulose-5-phosphate (Xu5P) but also on short-chain ketoses, converting them into AcP (Figs 2B and S3–S5 and S1 Table and S1 Data). Among all of the studied genes, PK from *Actinobacteria Bifidobacterium* (BbPK) displayed relatively high activity on all tested ketoses. We used quantum-chemical analysis to determine the catalytic processes of BbPK on short ketoses (S6–S11 Figs and S2–S4 Tables). Because 2-α, β-dihydroxyethylidene-ThDP (DHEThDP) is the intermediate of forming AcP from F6P or X5P by PKs, we calculated the formation process of DHEThDP from short ketoses. The energy profiles demonstrated feasibility of forming DHEThDP from short-chain ketoses through intramolecular proton transfer process (S8 Fig). However, the strong anchoring effect of the phosphate moiety of dihydroxyacetone phosphate (DHAP) and D-erythrulose-4-phosphate (Eu4P) led to the carbonyl groups being positioned far from the active C2 position of ThDP, reducing their interaction with ThDP to produce the covalent intermediate (S12 Fig).

**Fig 3:**
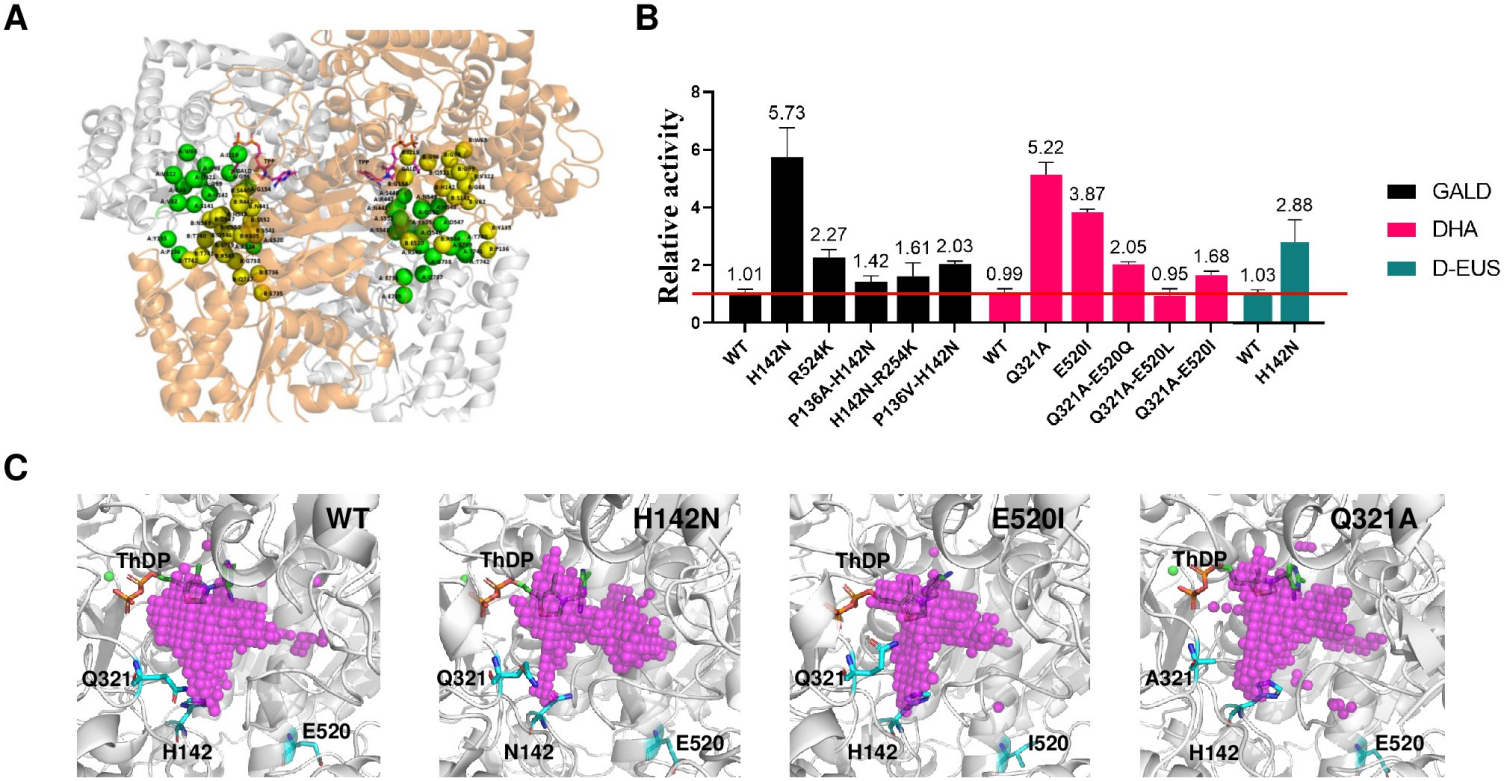
The directed evolution of BbPK. (**A**) The complex structure model of BbPK and GALD. The binding pockets of the substrates are situated at the interface of the BbPK homodimer. The 2 chains of the BbPK homodimer are distinguished by their white and orange colors. Residues comprising the pockets and key interfacial residues near the pockets are represented by green and yellow spheres based on their chain IDs. These residues are appropriately labeled with their chain ID and residue ID. (**B**) The relative activity of wild-type BbPK and beneficial mutants is assessed for GALD, DHA, and D-EUS. Relative activity was determined by calculating the ratio of the conversion rate of substrate for mutants to that of the wild-type. The raw data was listed in S1 Data. (**C**) The pocket volumes of WT, H142N, E521I, and Q321A. Pink dots represent the volumes of the binding pockets, which are measured as 366.6, 269.3, 254.5, and 346.6 Å^3^, respectively. The structures of the mutants were obtained through AlphaFold and further subjected to 50 ns molecular dynamics simulations. The figures were generated using Pymol software version 2.3.0, while the pockets were calculated using POVME 3.0. D-EUS, D-erythrulose; DHA, dihydroxyacetone; GALD, glycolaldehyde.

To further enhance the activity of BbPK on short-chain sugars, we performed directed evolution experiments. Since the formation process of DHEThDP from glycolaldehyde (GALD) and dihydroxyacetone (DHA) differs [20], we independently screened mutants with improved activities using 2 different high-throughput screening methods for GALD and DHA (S13–S16 Figs and S1 Data). Considering that the catalytic center of BbPK forms at the interface of a homodimer [17], we targeted residues located at the active cavity and protein-dimer interface for saturation mutagenesis (Fig 3A). Through screening around 3,060 mutants (S13 and S15 Figs), we identified potential sites that showed remarkable improvement in catalytic activity. For GALD, the potential residues were P136, H142, S440, R524, and S739. While for DHA, the potential residues were Q321, E520, G550, E735, and P136. Based on these potential activity-enhancing sites, we implemented random pairwise combinations and iterative saturation mutagenesis. After screening around 3,840 mutants (S14 and S16 Figs), the mutants exhibiting relative higher activities were subsequently sequenced and characterized (Figs 3B and S17 and S5 Table and S1 Data). Surprisingly, the combination mutations did not yield improved activities, and the best mutants were all single-point mutants. The dynamic parameters revealed that PK-E520I and PK-Q321A displayed 2.3-fold and 5-fold higher catalytic efficiency for DHA compared to wild-type BbPK, respectively (S17 Fig and S5 Table). Additionally, PK-H142N showed 8.5-fold higher catalytic efficiency for glycolaldehyde and 3.6-fold higher catalytic efficiency for D-erythrulose (D-EUS) than the wild-type BbPK (S17 Fig and S5 Table). Importantly, the 3 improved mutants exhibited smaller pocket volumes than wild-type BbPK, potentially increasing the likelihood of small substrates reaching a reactive state, which likely contributed to their enhanced activities (Fig 3C).

**Fig 4:**
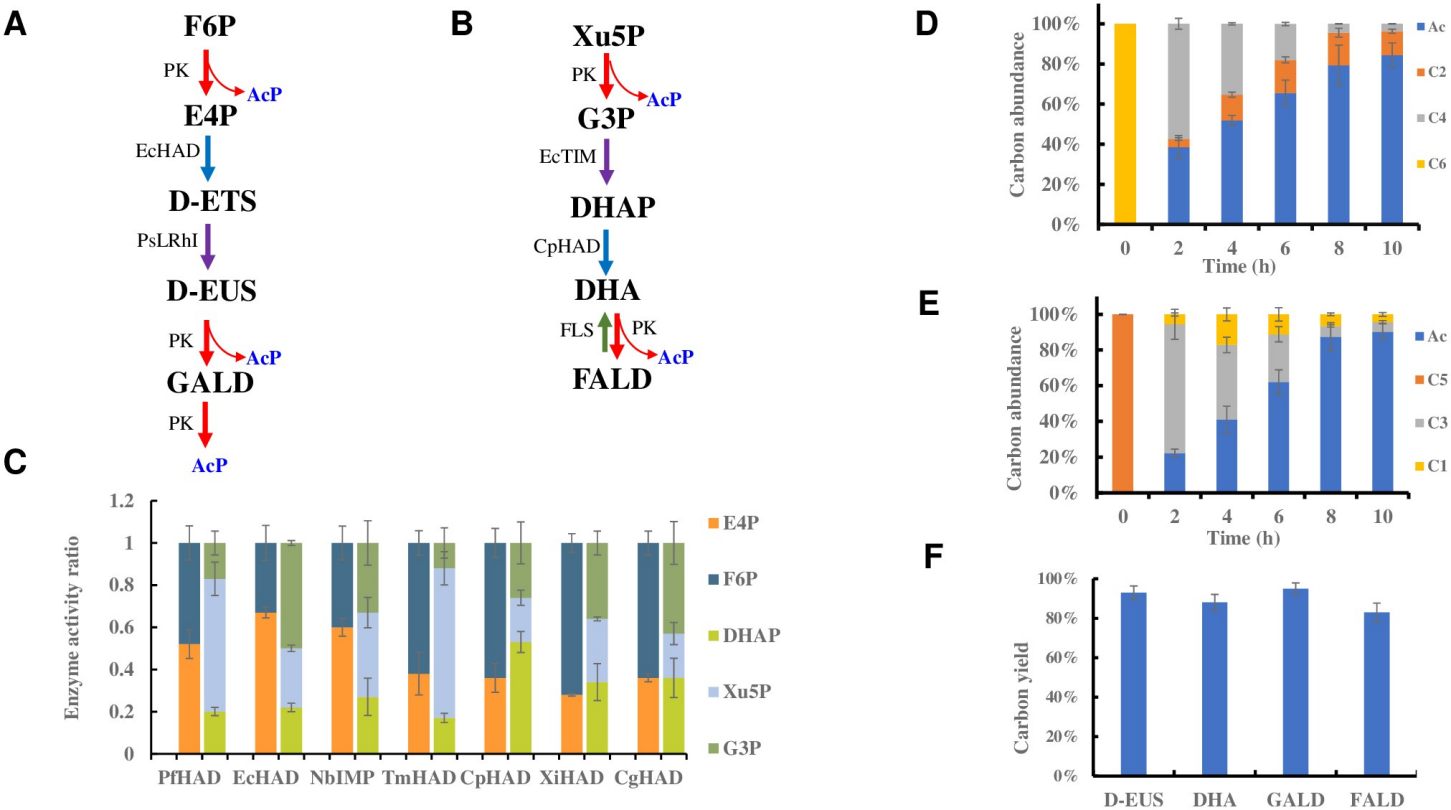
The APK pathway for utilization of multiple carbon sources in vitro. (**A**) The APK pathway for the synthesis of acetyl phosphate from F6P. F6P, fructose-6-phosphate; E4P, D-erythrose-4-phosphate; D-ETS, D-erythrose; D-EUS, D-erythrulose; GALD, glycolaldehyde. (**B**) The APK pathway for the synthesis of acetyl phosphate from Xu5P. Xu5P, D-xylulose-5-phosphate; G3P, D-glyceraldehyde-3-phosphate; DHAP, dihydroxyacetone phosphate; DHA, dihydroxyacetone; FALD, formaldehyde. (**C**) The screening process for specific phosphatases targeting E4P and DHAP. Enzyme activity refers to the conversion of substrates per unit time. Detailed information on phosphatases can be found in S8 and S9 Tables. (**D**) Metabolite analysis of the APK pathway for F6P. The initial concentration F6P was 10 mM. Ac, acetic acid; C2, glycoaldehyde; C4 refer to D-erythrose, D-erythrulose, and D-erythrose-4-phosphate. (**E**) Metabolite analysis of the APK pathway for Xu5P. The starting Xu5P concentration was 10 mM. C1 refer to formaldehyde, C3 refer to D-glyceraldehyde, dihydroxyacetone, dihydroxyacetone phosphate, and D-glyceraldehyde-3-phosphate. Carbon abundance refers to the ratio of carbon moles of an intermediate or product to the total carbon moles. (**F**) The APK pathway for the utilization of C1, C2, C3, and C4 carbon sources. The carbon yields from C1, C2, C3, and C4 carbon sources are 83%, 95%, 88%, and 93%, respectively. Error bars represent SD (standard deviation), *n* = 3. The raw data of Fig 4C–F was listed in S1 Data.

To validate the functionality of APK pathway based on BbPK, we decided to use the most abundant hexose D-glucose and pentose D-xylose as examples, which would be converted into F6P and Xu5P within the cells. Thus, F6P and Xu5P were selected as substrates for the study. BbPK catalyzed the conversion of F6P and Xu5P into AcP and D-erythorse-4-phosphate/glyceraldehyde-3-phosphate (E4P/G3P) [21]. Subsequently, 2 potential pathways for the further conversion of E4P/G3P into AcP were considered, taking into account the order of dephosphorylation and isomerization (S18 Fig). Considering the substrate promiscuity commonly observed in most phosphatases, it was deemed more efficient to reduce phosphorylated intermediates in the APK pathway. Therefore, E4P was first dephosphorylated and then isomerized into D-EUS (Fig 4A). On the other hand, if G3P were dephosphorylated first, there was no available enzyme to facilitate the isomerization of glyceraldehyde to DHA [22]. Hence, we proposed converting G3P into DHAP and subsequently producing DHA through the action of phosphatase, followed by the reaction catalyzed by BbPK (Fig 4B).

Considering the presence of multiple phosphate intermediates in the APK pathway and the limited selectivity of most phosphatases, it was necessary to identify specific phosphatases with high selectivity for E4P and DHAP. Seven phosphatases from different species were screened for this purpose (S19 Fig). Among them, HAD-like hydrolase (EcHAD) from *E*. *coli* displayed the highest specificity for E4P, with activity 2-fold higher than that on F6P (Fig 4C and S1 Data). Additionally, sugar phosphatase from *Candida parapsilosis* (CpHAD) displayed the highest specificity for DHAP, with activity more than 2-fold higher than that on Xu5P and G3P (Fig 4C and S1 Data). In addition, L-rhamnose isomerase (Ps-LRhI) from *pseudomonas stutzeri* was used to catalyze the isomerization of D-erythrose [23], and triose phosphate isomerase (EcTIM) from *E*. *coli* was used to isomerize G3P.

In addition, when dealing with ketoses with an even number of carbon atoms, they could be completely converted into a half the even number of AcP by PK and isomerase. However, ketoses with an odd number of carbon atoms would retain 1 molecule of formaldehyde at the end, resulting in carbon loss. To address this problem, we proposed the introduction of the formose reaction to condense formaldehyde into glycolaldehyde or DHA [20,24], which would then be further converted into AcP by BbPK (S20 Fig). The catalytic activities of BbPK on GALD and DHA were compared. Owing to the higher affinity of BbPK for DHA (S5 Table), formolase (FLS) was chosen to recycle formaldehyde in the system. Since AcP is unstable, we quantified AcP by converting AcP into acetic acid. With an input of 10 mM DHA, the residual formaldehyde in the system was consistently remained below 1 mM, and the yield of acetic acid reached 82% (S21 Fig and S1 Data), validating the recycling of most of formaldehyde generated in the APK pathway.

With all the key enzymes available, we successfully assembled the APK pathway in vitro. For the conversion of F6P, the APK pathway included BbPK, EcHAD, and PsLRhI, while for Xu5P, it involved BbPK, EcTIM, CpHAD, and FLS (Fig 4A and 4B). Under optimal reaction conditions, the final yields of acetic acid from F6P and Xu5P reached 84% and 90%, respectively (Fig 4D and 4E and S1 Data), surpassing the 67% yield of the glycolytic pathway [13]. Metabolite analysis showed that BbPK rapidly cleaved F6P and Xu5P within the first 2 h (Fig 4D and 4E). However, the conversion of E4P or G3P to AcP was slow due to the low catalytic efficiency of BbPK on GALD, DHA, and D-EUS (S5 Table). After 10 h, only small amounts of GALD and D-EUS remained in the F6P reaction system, along with traces of formaldehyde and DHA in the Xu5P reaction system. Furthermore, we tested the APK pathway for C1, C2, C3, and C4 carbon source in vitro (Figs 4F and S22 and S1 Data). The results demonstrated that the APK pathway enabled to achieve the nearly stoichiometric synthesis of acetyl-CoA from all tested carbon sources.

To demonstrate the potential of APK pathway in vivo, we performed growth assays of APK pathway in an engineered *E*. *coli* stain using glycerol as the carbon source. The *glpk* gene was knocked out to prevent the production of glycerol 3-phosphate from glycerol, while the *dhak* gene was knocked out to prevent the formation of DHAP from DHA. The promoter P*tac* was used to overexpress *pk* in the plasmid (S23 Fig). The results showed that the control strain MG1655Δ*glpk*Δ*dhak* could not grow on glycerol medium due to the knockout of the related metabolic pathway. However, the growth of the strain MG1655Δ*glpk*Δ*dhak* harboring the plasmid pBD-PK(SPK) was restored due to the conversion of glycerol to biomass through the APK pathway based on the catalytic activity of PK on DHA (S23 Fig and S1 Data). Although the difference in growth was only noticeable after 20 h due to the low activity of PK, and the growth of SPK was extremely weak, our results demonstrate the potential of APK pathway and its future used in constructing cell factories.

Depending on the versatile PK, it enables the conversion of various carbon sources to AcP and subsequent generation of acetyl-CoA. However, the low activities of BbPK on short-chain ketoses pose a major limitation. In fact, the standard Gibbs free energy change (ΔG’) of the BbPK for short-chain ketoses are all thermodynamically favorable, similar with Xu5P or F6P (S6 Table). The irreversible phosphorylytic cleavage of Xu5P or F6P by PK is both thermodynamically and kinetically favorable, which is one of the primary reasons why PK can shift the carbon flux and improve the carbon yield of acetyl-CoA derivatives in metabolic engineering [8,25–27]. Therefore, it is possible to improve the catalytic efficiency of BbPK for short-chain ketoses to enhance the ability of APK pathway for in vivo applications in the future. Hence, the APK pathway provides a simple approach to carbohydrate metabolism, offering advantages not only for utilizing of complex carbon sources but also in terms of atom economy compared to other pathways (S24 Fig and S7 Table). The design, construction, and test of APK pathway indicate the potential for using multiple carbon sources with higher efficiency in biomanufacturing in the future.

## Materials and methods

### Quantum-chemical analysis for PK from *Actinobacteria Bifidobacterium* (BbPK)

The computational model was obtained using AlphaFold [28]. The complex of BbPK with ThDP and substrate was generated with PyMOL [29]. The model contains 218 atoms with a total charge of +1, including the side chains of His64, His553, Glu479, Tyr501, Gln321, Ser440, His142, Gly155, His320, Asn549, Gln546, His548, His97, and the cofactor ThDP. Generally, the glutamate (Glu479) is modeled in protonated state for forming a hydrogen bond with the N1’ atom of ThDP. His97 was considered as the most possible candidate of proton donor for dehydration process, and it was modeled in the doubly protonated state. According to the interaction mode of pocket residues, His64, His553, His320, His142, His548, were modeled in their singly protonated states. The structural models of short-chain ketoses were obtained from the PubChem.

All the calculations were performed using the Gaussian 09 package [30] and the B3LYP method. The 6-31G (d, p) basis set was used for the geometry optimizations, and the electronic energies of the stationary points were refined by single-point calculations with the 6–311++G (2d, 2p) basis set. Solvation energies were calculated with the SMD [31] implicit solvent method and a dielectric constant of ε = 4. Previous studies have shown that the effect of the solvation diminishes rapidly with the size of the active site model, rendering the particular value used for the dielectric constant less critical. The zero-point energy corrections were done at the same level of theory as the geometry optimizations.

As used in the cluster approach [32,33], a number of atoms were kept fixed to their crystallographic positions in the geometry optimizations (indicated by asterisks in the S6 Fig). This coordinate-fixing protocol is very important to avoid large unrealistic movements of the various groups at the active site. This approach deals only with the chemical steps of the enzymatic reactions, implying that the substrate binding and product release are usually not explicitly considered by the calculations. Therefore, an implicit assumption in the current model is that neither of these events are rate- or selectivity-determining.

### Molecular docking of substrate to BbPK

The molecular docking tool of GNINA [34] was used to predict the potential interaction of different ligands (DHAP, Eu4P, Xu5P, F6P, D/L-EUS, and DHA) with PK from *Actinobacteria Bifidobacterium*. A structural model of BbPK was obtained using AlphaFold [28] and a 20-nanosecond molecular dynamics simulation was performed to optimize the side chain conformations. The refined PK structural model was treated as the receptor for docking. The structural models of the ligands and ThDP were obtained from the PubChem. The binding site was determined by the structural alignment to the crystal structure of a known phosphoketolase (PDB ID:3AHE) [15]. The docking models were ranked by GNINA’s CNN pose score. For each ligand, the top 1 docking model was selected and energy minimization was performed by OpenMM7 [35] with the Amber14 [36] force field to optimize the complex model.

### Phosphoketolase selection

To investigate the function of PK genes in distant evolutionary branches, we screened and analyzed all potential PKs in the NCBI database. First, we predicted all potential PKs by search against the nonredundant database with the Pfam domain ID PF03894 (hmmscan—cpu 10—domtblout output.txt -E 1e-4 PF03894.hmm NR.fasta) [37]. Second, we retrieved all PKs from KEGG database (https://www.genome.jp/entry/pf:xfp). Third, we made a local blastp search using PKs from NCBI as the query sequences, and PKs from KEGG as the BLAST database. After blastp search, 12,185 potential PKs were screened with 3 standards: the best hit is a D-xylulose 5-phosphate/D-fructose 6-phosphate phosphoketolase (XFP, EC:4.1.2.9 4.1.2.22, KO: K01621), the identity is more than 40, and the align length is more than 600. Fourth, all PKs were classified into 4,101 groups by using OrthoMCL with the amino acid identity more than 90 in a group. For each group, we selected a PK gene, which is closest approximation to supposed optimal sequence, consisting of the highest frequency residues in multiple sequences alignment. Using the similar strategy, 23 PKs were screened based on the standard of the identity of 60.

### Protein engineering of BbPK

To obtain full mutations, oligonucleotide primers were designed with the degenerate codon NNK, which cover almost all mutations with only 96 clones. Hence, a total of 3,264 clones were screened against 34 single-site saturation mutation libraries. Each single-site saturation mutant library was generated based on PCR. The PCR product was degraded the template with DpnI restriction endonuclease, and then transformed into *E*. *coli* BL21 (DE3) competent cells for library construction. Each colony was incubated in 200 μL of LB medium for 24 h to plateau at 37°C and then transferred to 1 mL of the same medium for protein expression. The cells, which induced by IPTG (isopropyl-β-D-thiogalactopyranoside) and cultured overnight at 16°C, were harvested by centrifugation. The bacterial pellet was washed and resuspended in reaction buffer (50 mM potassium phosphate buffer, 5 mM GALD or 20 mM DHA (pH 7.4)). After 3 h of reaction at 37°C, the supernatant was collected by centrifugation for detection of substrate or product.

For GALD, we determined the activity of the mutants by detecting the reduction of substrate. The detection method was as follows: added 120 μL chromogenic reagent to 60 μL sample and heated at 90°C for 15 min. Subsequently, the residual substrate concentration was measured spectrophotometrically at 650 nm. The chromogenic reagent: 1.5 g diphenylamine was dissolved in 100 mL acetic acid, and then 1.5 mL pure sulfuric acid was added.

For DHA, we screened for highly active mutants by detecting the product formaldehyde. The formaldehyde detection method was as follows: 40 μL sample was mixed with 160 μL chromogenic reagent, and then heated at 60°C for 10 min. Subsequently, formaldehyde production was measured spectrophotometrically at 440 nm, and 100 mL chromogenic reagent (pH 6.0) contains 25 g ammonium acetate, 3 mL acetic acid, and 0.25 mL acetylacetone solution.

### Activity assay and kinetic properties of PK and mutants

The standard reaction mixture (100 μL) contained 50 mM potassium phosphate buffer (pH 7.5), 5 mM MgSO_4_, 1 mM ThDP, 10 mM GALD (DHA or D-EUS), 1 mM ADP, 0.2 mg mL^−1^ AckA, 5 U hexokinase, 2.5 U Glucose-6-phosphate dehydrogenase, 1 mM NADP^+^, and 10 mM glucose. Approximately 0.5 mg mL^−1^ PK was added into the reaction system. The reactions conducted at 37°C. NADPH was detected spectrophotometrically at 340 nm. Enzyme kinetics with GALD (DHA or D-EUS) as substrate were determined in assays with GALD (DHA or D-EUS) concentrations of 0–110 mM. Kinetic parameters kcat and Km were determined by measuring the initial velocities of the enzymic reaction and curve-fitting according to the Michaelis–Menten equation, using GraphPad Prism 5 software.

### Bacterial strains and growth condition

*Escherichia coli DH5α* strain (TransGenTM) was grown at 37°C in LB medium for gene cloning and other DNA manipulations. *E*. *coli BL21 (DE3)* (TransGenTM) was grown at 37°C or 16°C in 2YT medium for protein expression. Antibiotics for selection purposes were used with 100 μg mL^−1^ spectinomycin, 100 μg mL^−1^ ampicillin, 34 μg mL^−1^ chloramphenicol, or 100 μg mL^−1^ kanamycin.

### Enzyme activity of BbPK for DHA, L-EUS, and D-EUS

The coding gene of phosphoketolase from *Actinobacteria Bifidobacterium* was ligated into the expression vector pET-28a via NdeІ and XhoI restriction sites. *E*. *coli* BL21(DE3) cells carrying recombinant plasmid were inoculated into 5 mL LB (Luria Broth) medium with Kanamycine (100 μg mL^−1^) and cultured overnight at 37°C, and then scaled up to 800 mL 2YT medium (16 g L^−1^ Tryptone, 10 g L^−1^ yeast extract, 5 g L^−1^ NaCl) containing Kanamycine (100 μg mL^−1^). Gene expression was induced by adding IPTG to a final concentration of 0.5 mM when OD_600_ reached 0.6. The cell cultures continued to grow overnight at 16°C before being harvested by centrifugation at 6,000×g and then was resuspended in 50 mL lysis buffer (50 mM potassium phosphate buffer (pH 7.4), 5 mM MgSO_4_, 0.5 mM ThDP). The bacterial pellet was lysed by using a high-pressure homogenizer (JNBIO, China), and the cell debris was removed by centrifugation at 10,000×g for 60 min at 4°C. The soluble protein sample was loaded onto a nickel affinity column (GE Healthcare), which was rinsed with 50 mL wash buffer (50 mM potassium phosphate buffer (pH 7.4), 5 mM MgSO_4_, 0.5 mM ThDP, and 50 mM imidazole) and then eluted with 20 mL elution buffer (50 mM potassium phosphate buffer (pH 7.4), 5 mM MgSO_4_, 0.5 mM ThDP, and 200 mM imidazole). The eluted protein was concentrated and dialyzed against lysis buffer (50 mM potassium phosphate buffer (pH 7.4), 5 mM MgSO_4_, and 0.5 mM ThDP) by ultrafiltration with an Amicon Ultra centrifugal filter device (Millipore, USA) with a 30 kDa molecular-weight cutoff. The protein concentration was determined using a BCA Protein Assay Reagent Kit (Pierce, USA) with BSA as the standard.

Activity of PKs on DHA was determined with 1 mg mL^−1^ purified recombinant protein in 200 μL reaction mixtures. The reaction system comprised 10 mM DHA, 1 mM ThDP, 5 mM MgSO_4_, and 50 mM phosphate buffer (pH 7.4). After incubation at 37°C for 0.5 h, the reaction was stopped by adding 200 μL of acetonitrile. Acetyl-phosphate in samples is determined by chromogenic and liquid chromatography, the by-product formaldehyde was confirmed by GC-MS.

Chromogenic detection of acetyl-phosphate (AcP): add 100 μL hydroxylamine hydrochloride solution (2 M, pH 6.5) to 100 μL sample, conduct at 30°C for 10 min. Then add 200 μL of chromogenic solution, which is prepared with 10 mL FeCl_3_.6H_2_O (FeCl_3_.6H_2_O powder dissolved in 0.1 M hydrochloric acid, 5% (m/v)), 10 mL trichloroacetic acid (15% (m/v)), and 10 mL HCl (4 M), conduct at 30°C for 5 min, then detect the absorbance at 505 nm.

HPLC detection for AcP: add equal volume of 5% sulfuric acid to the sample for completely decomposing AcP into acetic acid. Acetic acid was then detected by HPLC. HPLC conditions: column, Aminex HPX-87H (Bio-Rad); detection wavelength, 210 nm; mobile phase, 5 mM sulfuric acid; flow rate, 0.6 mL min^−1^; sample volume, 20 μL; column temperature, 40°C.

Formaldehyde detection by GC-MS. Sample derivatization: 100 μL 2.4-dinitrophenylhydrazine solution was added to 100 μL samples, the mixture was performed at 60°C for 60 min in the dark. Then added 400 μL of n-hexane to the mixed solution, centrifuge at 5,000×g for 2 min. Separated the upper solution and then added appropriate amount of anhydrous sodium sulfate powder, centrifuged at 10,000×g for 10 min. Separated the upper solution and was detected by GC-MS. GC-MS conditions: Electron ionization (EI) GC-MS analyses were performed with a model 7890A GC (Agilent) with a DB-5 fused silica capillary column (30 cm length, 0.25 mm inner diameter, 0.25 μm film thickness) coupled to an Agilent 7200 Q-TOF mass selective detector. Injections were performed by a model 7683B autosampler. The GC oven was programmed from 160°C (held for 1 min) to 240°C at 10°C min^−1^, and then held for 5 min; the injection port temperature was 250°C, and the transfer line temperature was 280°C. The carrier gas, ultra-high purity helium, flowed at a constant rate of 1.2 mL min^−1^. For full-scan data acquisition, the MS scanned from 35 to 550 atomic mass units. Data analysis for GC-MS was performed with Mass Hunter software (Agilent, USA) and NIST Database.

The activity of PKs on L-EUS and D-EUS were determined with 1 mg mL^−1^ purified recombinant protein in 200 μL reaction mixtures. The reaction system comprised 10 mM L-EUS or D-EUS, 1 mM ThDP, 5 mM MgSO_4_, and 50 mM phosphate buffer (pH 7.4). After incubation at 37°C for 0.5 h, the reaction was stopped by adding 200 μL of acetonitrile. AcP in samples was detected by chromogenic and liquid chromatography. The by-product glycoaldehyde was confirmed by GC-MS. Chromogenic and liquid chromatography detection of AcP were same as DHA.

Glycoaldehyde was confirmed by GC-MS. Samples were freeze-dried, then 60 μL of 0.2 M PFBOA solution was added and mixed, the reaction was performed at 30°C for 90 min. A total of 300 μL of n-hexane was added and centrifuge at 5,000×g for 2 min. Separated the upper solution and then added appropriate amount of anhydrous sodium sulfate powder, centrifuged at 10,000×g for 10 min. Separated 100 μL upper solution, then 30 μL N-Methyl-N-(trimethylsilyl)-trifluoroacetamide with 1% trimethylchlorosilane was added and mixed, the reaction system conducted at 30°C for 30 min. The product was detected by GC-MS. GC-MS conditions: EI GC-MS analyses were performed with a model 7890A GC (Agilent) with a DB-5 fused silica capillary column (30 cm length, 0.25 mm inner diameter, 0.25 μm film thickness) coupled to an Agilent 7200 Q-TOF mass selective detector. Injections were performed by a model 7683B autosampler. The GC oven was programmed from 60°C (held for 1 min) to 100°C at 5°C min^−1^, to 300°C at 25°C min^−1^ and then held for 5 min; the injection port temperature was 250°C, and the transfer line temperature was 280°C. The carrier gas, ultra-high purity helium, flowed at a constant rate of 1.2 mL min^−1^. For full-scan data acquisition, the MS scanned from 35 to 550 atomic mass units. Data analysis for GC-MS was performed with Mass Hunter software (Agilent, USA) and NIST Database.

Activity of PKs on E4P, D-ETS, D-GCD, and DHAP were determined with 1 mg mL^−1^ of purified recombinant protein in 200 μL reaction mixtures. The reaction system included substrate (10 mM E4P, D-ETS, D-GCD, or DHAP), 1 mM ThDP, 5 mM MgSO_4_, and 50 mM phosphate buffer (pH 7.4). After incubation at 37°C for 0.5 h, the reaction was stopped by adding 200 μL of acetonitrile. AcP in samples was detected by chromogenic and liquid chromatography. Chromogenic and liquid chromatography detection of AcP were same as DHA.

Activity of PKs on erythrulose-4-phosphate (Eu4P) were determined with 1 mg mL^−1^ purified recombinant protein in 200 μL reaction mixtures. The reaction system included 10 mM Eu4P, 0.5 mg mL^−1^ RpiB, 1 mM ThDP, 5 mM MgSO_4_, and 50 mM phosphate buffer (pH 7.4). After incubation at 37°C for 0.5 h, the reaction was stopped by adding 200 μL of acetonitrile. AcP in samples was detected by chromogenic and liquid chromatography. Chromogenic and liquid chromatography detection of AcP were same as DHA.

### Expression, purification, and enzyme kinetics of PKs from different species

The PKs coding genes from different species were ligated into the expression vector pET-28a via *Nde*І and *Xho*I restriction sites. All genes were expressed in BL21 (DE3) and purified on the Ni-NTA column. Large-scale purification (800 mL) typically produced about 50 mg enzyme. The protein concentration was determined using the BCA Protein Assay Reagent Kit (Pierce, USA) with BSA as the standard.

Determination of kinetics of PKs on GALD, DHA, L-EUS, and D-EUS. The standard reaction mixture (200 μL) contained 50 mM potassium phosphate buffer (pH 7.5), 5 mM MgSO_4_, 1 mM ThDP, 10 mM GALD (or, DHA, L-EUS, D-EUS), 1 mM ADP, 0.2 mg mL^−1^ AckA, 1 U hexokinase, 0.5 U glucose-6-phosphate dehydrogenase, 1 mM NADP^+^, and 10 mM glucose. Various PKs (0.25 mg mL^−1^) from different species were added into the reaction system. The reactions conducted at 37°C. The production of NADPH was detected at 340 nm. Enzyme kinetics with DHA, L-EUS, and D-EUS as substrates were determined in assays with concentrations of 0–110 mM. Kinetic parameters were determined from triplicate experiments using GraphPad Prism 5 (GraphPad Software, USA).

Determination of kinetics of PKs on Xu5P. The standard reaction mixture (200 μL) contained 50 mM potassium phosphate buffer (pH 7.5), 5 mM MgSO_4_, 1 mM ThDP, 5 mM Xu5P, 0.05 mg mL^−1^ TIM, 1 U glycerophosphate dehydrogenase, and 1 mM NAD^+^. Various PKs (0.1 mg mL^−1^) from different species were added into the reaction system. The reactions conducted at 37°C. The production of NADH was detected at 340 nm.

Determination of kinetics of PKs on F6P. The standard reaction mixture (200 μL) contained 50 mM potassium phosphate buffer (pH 7.5), 5 mM MgSO_4_, 1 mM ThDP, 5 mM F6P, 0.3 mg mL^−1^ erythrose-4-phosphate dehydrogenase, and 1 mM NAD^+^. Various PKs (0.1 mg mL^−1^) from different species were added into the reaction system. The reactions conducted at 37°C. The production of NADPH was detected at 340 nm.

### Expression, purification, and enzyme kinetics of phosphatases from different species

The phosphatases coding genes of different species were ligated into the expression vector pET-28a via NdeІ and XhoI restriction sites. All genes were expressed in BL21 (DE3) and purified on the Ni-NTA column. Large-scale purification (800 mL) typically produced about 5 to 50 mg enzyme. The protein concentration was determined using a BCA Protein Assay Reagent Kit (Pierce, USA) with BSA as the standard.

The kinetics of phosphatases on F6P, Xu5P, E4P, G3P, and DHAP were determined by monitoring the production of fructose, xylulose, ETS, GCD, and DHA by HPLC. The standard reaction mixture (100 μL) contained 50 mM potassium phosphate buffer (pH 7.5), 5 mM F6P (or Xu5P, E4P, G3P, DHAP). Various phosphatases (0.1 mg mL^−1^) from different species were added into the reaction system. The reactions conducted at 37°C for 0.5 to 2 h, and 100 μL acetonitrile was added to the samples to terminate the reactions, and then analyzed by HPLC.

HPLC condition for GCD or DHA: column, Aminex HPX-87H (Bio-Rad); detection wavelength, 210 nm; mobile phase, 5 mM sulfuric acid; flow rate, 0.6 mL min^−1^; sample volume, 20 μL; column temperature, 40°C.

HPLC detection for fructose, xylulose, and erythrose. Sample derivatization: Reaction samples (50 μL) were mixed with a solution of O-benzylhydroxylamine hydrochloride (50 μL, 0.14 mmol mL^−1^) (pyridine: methanol: water = 33:15:2). After incubation at 50°C for 60 min, samples were diluted in methanol (100 μL) and directly analyzed by HPLC chromatography. HPLC condition: column, X-BridgeTM C18, 5 μm, 4.6 × 250 mm column from Waters (Milford, USA); sample volume, 30 μL; solvent system, (A) aqueous trifluoroacetic acid (TFA) (0.1% (v/v) and (B) TFA (0.095% (v/v)) in CH_3_CN/H_2_O (4:1), gradient elution from 20% to 60% B in 16 min; flow rate, 1 mL min^−1^; detection wavelength, 215 nm; column temperature, 35°C.

### Formaldehyde circulation system verification

Comparison of PK-GALS formaldehyde recycling system and PK-FLS formaldehyde recycling system: The 100 μL reaction system contained 50 mM potassium phosphate buffer (pH 7.5), 5 mM MgSO_4_, 2 mg mL^−1^ PK, 2 mg mL^−1^ GALS or FLS, 10 mM DHA, and 1 mM ThDP. The reactions conducted at 37°C for 2 h, and 100 μL sulfuric acid (5%) was added to the samples to terminate the reactions. Acetic acid was then detected by HPLC. HPLC conditions: column, Aminex HPX-87H (Bio-Rad); detection wavelength, 210 nm; mobile phase, 5 mM sulfuric acid; flow rate, 0.6 mL min^−1^; sample volume, 20 μL; column temperature, 40°C.

Acetic acid and DHA detection method is the same as above. Formaldehyde was detected by chromogenic method. Chromogenic detection of formaldehyde is as follows: dilute the sample to the appropriate concentration (0.1 to 1 mM), add 80 μL chromogenic solution to 120 μL of the diluted sample. The reaction conducted at 60°C for 10 min and centrifugal at 12,000×g for 5 min. Take 150 μL to measure the absorbance at 414 nm. Calculate the concentration of formaldehyde in the sample according to the standard curve. Chromogenic solution is prepared as follows: Dissolve 250 g of ammonium acetate in 900 mL of ddH_2_O, then add 30 mL of acetic acid and 2.5 mL of acetylacetone, adjust the pH of the solution to 6 with acetic acid, and finally add ddH_2_O to a volume of 1 L.

### Process analysis of the APK pathway for F6P

The 1 mL reaction system contained 50 mM potassium phosphate buffer (pH 7.5) 5 mM MgSO_4_, 2 mg mL^−1^ PK, 0.5 mg mL^−1^ EcHAD, 1 mg mL^−1^ Ps-LRHI, 1 mM ThDP, 10 mM F6P. The reactions conducted at 37°C for 10 h. Samples were taken out every 2 h for analysis. The reactions were terminated by heating at 95°C for 5 min, and then cooled down to 37°C. Added the 5 U alkaline phosphatase, and conducted at 37°C for 4 h, equal volume acetonitrile was used to terminate the reaction. Fructose, D-ETS, D-EUS, GALD, and acetic acid were detected by HPLC.

Sample derivatization: Reaction samples (50 μL) were mixed with a solution of O-benzylhydroxylamine hydrochloride (50 μL, 0.14 mmol mL^−1^) (pyridine: methanol: water = 33:15:2). After incubation at 50°C for 60 min, samples were diluted in methanol (100 μL) and directly analyzed by HPLC. HPLC conditions: column, X-Bridge TM C18, 5 μm, 4.6 × 250 mm column from Waters (Milford, USA); sample volume, 30 μL; mobile phase, solvent system (A) TFA (0.1% (v/v) and (B) TFA (0.095% (v/v)) in CH_3_CN/H_2_O (4:1), gradient elution from 20% to 60% B in 16 min; flow rate, 1 mL min^−1^; detection wavelength, 215 nm; column temperature, 35°C.

### Process analysis of the APK pathway for Xu5P

The 1 mL reaction system contained 50 mM potassium phosphate buffer (pH 7.5) 5 mM MgSO_4_, 2 mg mL^−1^ PK, 1 mg mL^−1^ CpHAD, 0.1 mg mL^−1^ TIM, 2 mg mL^−1^ FLS, 1 mM ThDP, and 10 mM Xu5P. The reactions conducted at 37°C for 10 h, and samples were taken out every 2 h for analysis. The reactions were terminated by heating at 95°C for 5 min, then cooled down to 37°C. Added the 5 U alkaline phosphatase, and conducted at 37°C for 4 h, equal volume acetonitrile was used to terminate the reaction. D-xylulose, GCD, DHA, and acetic acid were detected by HPLC. Formaldehyde was detected by chromogenic method as before.

HPLC detection for D-xylulose, GCD and DHA. Sample derivatization: Reaction samples (50 μL) were mixed with a solution of O-benzylhydroxylamine hydrochloride (50 μL, 0.14 mmol mL^−1^) (pyridine: methanol: water = 33:15:2). After incubation at 50°C for 60 min, samples were diluted in methanol (100 μL) and directly analyzed by HPLC chromatography. HPLC conditions: column, X-BridgeTM C18, 5 μm, 4.6 × 250 mm column from Waters (Milford, USA); sample volume, 30 μL; mobile phase, solvent system (A) TFA (0.1% (v/v) and (B) TFA (0.095% (v/v)) in CH_3_CN/H_2_O (4:1), gradient elution from 20% to 60% B in 16 min; flow rate, 1 mL min^−1^; detection wavelength, 215 nm; column temperature, 35°C. HPLC conditions of acetic acid were the same as before.

### The APK pathway for the utilization of C1-C4 carbon sources

The APK pathway for D-EUS and GALD. The 200 μL reaction system contained 50 mM potassium phosphate buffer (pH 7.5), 5 mM MgSO_4_, 2 mg mL^−1^ PK, 1 mM ThDP, 10 mM GALD, or 5 mM D-EUS. The reactions conducted at 37°C overnight.

The APK pathway for FALD and DHA. The 200 μL reaction system contained 50 mM potassium phosphate buffer (pH 7.5), 5 mM MgSO_4_, 2 mg mL^−1^ PK, 2 mg mL^−1^ FLS, 1 mM ThDP, 20 mM FALD, or 10 mM DHA. The reactions conducted at 37°C overnight.

## Supporting information

S1 Fig The phosphoketolase (PK) pathway in nature. Fructose-6-phosphate (F6P) and xylulose-5-phosphate (Xu5P) are converted into D-erythorse-4-phosphate/glyceraldehyde-3-phosphate (E4P/G3P) and acetyl-phosphate (AcP), which not only can be converted into acetyl-CoA by phosphate acetyltransferase (PTA), but also can be converted to ATP and acetate by acetate kinase (AK).
(TIF)

S2 Fig The phylogenetic tree of PKs. “]” indicates that the species has been selected to test. “unex” indicates that the protein is not expressed correctly in *E*. *coli*. “fun” indicates that PKs have activities on F6P or Xu5P.
(TIF)

S3 Fig The activity test of PK from *Actinobacteria Bifidobacterium* (BbPK) on different sugars. The product acetyl phosphate was converted to acetic acid, which was detected by HPLC. The reaction system without PK was used as the control. L-EUS, L-erythrulose; D-EUS, D-erythrulose; Eu4P, D-erythrulose-4-phosphate; ETS, D-erythrose; DHA, dihydroxyacetone; GCD, D-glyceraldehyde; DHAP, dihydroxyacetone phosphate; F6P, D-fructose-6-phosphate.
(TIF)

S4 Fig The structure of tested substrates. (TIF)

S5 Fig Detection of formaldehyde and glycoaldehyde in PK- catalyzed reaction system. (**A**) Schematic illustration of the reaction of dihydroxyacetone, D-erythrulose, and L-erythrulose catalyzed by PK. (**B**) Formaldehyde was detected by GC-MS in PK-catalyzed dihydroxyacetone reaction system. (**C**) Glycoaldehyde was detected by GC-MS in PK-catalyzed D-erythrulose reaction system. (**D**) Glycoaldehyde was detected by GC-MS in PK-catalyzed L-erythrulose reaction system. Sample derivatization methods, see Materials and methods.
(TIF)

S6 Fig Computational model for calculation. The model contains 218 atoms with a total charge of +1, including the side chains of His64, His553, Glu479, Tyr501, Gln321, Ser440, His142, Gly155, His320, Asn549, Gln546, His548, His97, and the cofactor ThDP. The fixed atoms are labeled by asterisks.
(TIF)

S7 Fig Proposed catalytic mechanism of PK. (**A**) The forming process of 2-α, β-dihydroxyethylidene-ThDP (DHEThDP) (int3) from 1,3-dihydroxyacetone. (**B**) The forming process of DHEThDP from D-erythrulose. (**C**) The forming process of DHEThDP from L-erythrulose. Upon binding of the substrate to ThDP, the first step is a C−C bond formation that leads to an alkoxide tetrahedral intermediate. Next, an intramolecular proton transfer takes place from the hydroxyl group in C3 to the alkoxide. The last step is a C−C bond cleavage to form the DHEThDP. R, reactant; int1, intermediate 1; TS1, transition state 1.
(TIF)

S8 Fig Calculated energy profiles for the forming process of 2-α, β-dihydroxyethylidene-ThDP (DHEThDP) from short-chain ketoses. (**A**) The energy profiles for 1,3-dihydroxyacetone. (**B**) The energy profiles for D-erythrulose. (**C**) The energy profiles for L-erythrulose. Energies are given in kilocalories per mole. Note: After adding the large basis set, solvation, and zero-point energy corrections, the energies of TS2 for 1, 3-dihydroxyacetone and D-erythrulose were calculated to be lower than those of int1. Therefore, intramolecular proton transfer of 1,3-dihydroxyacetone and D-erythrulose can be assumed to be barrierless or to occur with very low barriers.
(TIF)

S9 Fig Optimized structures of the transition states and intermediate involved in the forming process of DHEThDP from 1,3-dihydroxyacetone. The key bond distances change is shown in the figure. Selected distances are given in Å. The distances between C2 of ThDP and carbonyl C of substrate changes from 3.1 Å in Reactant (R) to 2.2 Å in transition state 1 (TS1). The distance between O and H changes from 1.6 Å in intermediate 1 (int1) to 1.3 Å in TS2. The distance of C2 and C3 of substrate changes from 2.1 Å in TS3 to 2.8 Å in int3.
(TIF)

S10 Fig Optimized structures of the transition states and intermediate involved in the forming process of DHEThDP from D-erythrulose. The key bond distances change is shown in the figure. Selected distances are given in Å. The distances between C2 of ThDP and carbonyl C of substrate changes from 3.1 Å in Reactant (R) to 2.2 Å in transition state 1 (TS1). The distance between O and H changes from 1.6 Å in intermediate 1 (int1) to 1.2 Å in TS2. The distance of C2 and C3 of substrate changes from 2.1 Å in TS3 to 4.2 Å in int3.
(TIF)

S11 Fig Optimized structures of the transition states and intermediate involved in the forming process of DHEThDP from L-erythrulose. The key bond distances change is shown in the figure. Selected distances are given in Å. The distances between C2 of ThDP and carbonyl C of substrate changes from 3.1 Å in Reactant (R) to 2.1 Å in transition state 1 (TS1). The distance between O and H changes from 1.6 Å in intermediate 1 (int1) to 1.3 Å in TS2. The distance of C2 and C3 of substrate changes from 2.1 Å in TS3 to 4.4 Å in int3.
(TIF)

S12 Fig The docking results of different substrates in PK. The PK was shown in cartoon and colored gray. The ThDP, ligands, and 2 key residues R442 and K605 were shown in stick. The C, N, O, P, and S atoms were colored green, blue, red, orange, and yellow, respectively. The distance between the substrates and ThDP and the distances between the phosphate moiety of substrates and the key basic residues were shown as dashed lines. Selected distances are given in Å.
(TIF)

S13 Fig High-throughput screening of BbPK for glycolaldehyde (GALD). The x-axis labels represent the selected location in the BbPK. The y-axis labels represent the relative catalytic activities of the different mutants. Relative activity was defined as the ratio of the reduction of substrate for mutants to that of the wild type. The raw data was listed in S1 Data.
(TIF)

S14 Fig Iterative saturation mutagenesis of BbPK for glycolaldehyde (GALD). The x-axis labels represent the location combinations in the BbPK. The y-axis labels represent the relative catalytic activities of the different mutants. Relative activity was defined as the ratio of the reduction of substrate for mutants to that of the wild type. The raw data was listed in S1 Data.
(TIF)

S15 Fig High-throughput screening of BbPK for dihydroxyacetone (DHA). The x-axis labels represent the selected location in the BbPK. The y-axis labels represent the relative catalytic activities of the different mutants. Relative activity was defined as the ratio of the titer of formaldehyde for the mutants to that of the wild type. The raw data was listed in S1 Data.
(TIF)

S16 Fig Iterative saturation mutagenesis of BbPK for dihydroxyacetone (DHA). The x-axis labels represent the location combinations in the BbPK. The y-axis labels represent the relative catalytic activities of the different mutants. Relative activity was defined as the ratio of the titer of formaldehyde for the mutants to that of the wild type. The raw data was listed in S1 Data.
(TIF)

S17 Fig Enzyme kinetics of wild-type BbPK and mutants for different substrates. Enzyme kinetics were determined with 0.25 mg mL^−1^ enzyme. The concentration of substrate ranged from 1 to 110 mM. Error bars represent SD (standard deviation), *n* = 3. The raw data was listed in S1 Data.
(TIF)

S18 Fig Two different pathways for converting E4P/G3P to EUS/DHA. (**A**) The 2 conversion pathways of D-erythrulose (D-EUS) from D-erythrose-4-phosphate (E4P). (**B**) The 2 conversion pathways of dihydroxyacetone (DHA) from D-glyceraldehyde-3-phosphate (G3P). Dashed arrows represent the pathway that is first isomerized and then dephosphorylated. Solid arrows represent the pathway that is first dephosphorylated and then isomerized.
(TIF)

S19 Fig SDS-PAGE analysis of phosphatases from different species. (A), pfHAD; (B), EcHAD; (C), NbIMP; (D), TmHAD; (E), CpHAD; (F), XiHAD; (G), CgHAD. The detailed information of phosphatases, see S8 and S9 Tables.
(TIF)

S20 Fig Two different pathways to recycle formaldehyde. (**A**) Formaldehyde is converted to glycolaldehyde by glycolaldehyde synthase (GALS). Glycolaldehyde is then converted to AcP by PK. (**B**) Formaldehyde is converted to dihydroxyacetone by formolase (FLS). Dihydroxyacetone is then converted to AcP by PK.
(TIF)

S21 Fig Recycle the formaldehyde generated in the APK pathway. (**A**) The conversion of DHA to AcP using only BbPK in vitro. (**B**) Formolase (FLS) was used to recycle formaldehyde. The red curve represents the change of acetic acid concentration. The blue curve represents the change of DHA concentration. The gray curve represents the change of formaldehyde concentration. Acetic acid was detected by HPLC. Error bars represent SD (standard deviation), *n* = 3. The raw data was listed in S1 Data.
(TIF)

S22 Fig The APK pathway for the utilization of C1, C2, C3, and C4 carbon sources. (**A**) Methanol is converted to DHA and then converted to AcP via APK pathway. MDH, methanol dehydrogenase. (**B**) Ethylene glycol or ethanolamine is converted to glycoaldehyde and then converted to AcP via APK pathway. EGDH, ethylene glycol dehydrogenase; EOX, ethanolamine oxidase. (**C**) Glycerol is converted to DHA and then converted to AcP via APK pathway. GDH, glycerol dehydrogenase. (**D**) Erythritol is converted to erythrulose and then converted to AcP via APK pathway. ERDH, erythritol dehydrogenase. Dashed arrows represent the process that is not tested.
(TIF)

S23 Fig The implementation of APK pathway in vivo. (**A**) Schematic representation of the natural glycerol metabolic pathways and APK pathway. Red crosses represent gene knockout. Blue arrows represent APK pathway. The promoter P*tac* was used to overexpress *pk* in the plasmid. Metabolite abbreviation: DHA, dihydroxyacetone; DHAP, dihydroxyacetone phosphate; AcP, acetyl phosphate. Genes involved: glpk, glycerol kinase; dhak, dihydroxyacetone kinase. (**B**) Growth curve in glycerol minimal medium. The control was MG1655Δ*glpk*Δ*dhak*. The strain PK (SPK) was MG1655Δ*glpk*Δ*dhak* harboring plasmid pBD-PK. Plasmid pBD-PK was constructed for the expression of *pk* under the control of the Ptac promoter. Error bars represent SD (standard deviation), *n* = 3. The raw data was listed in S1 Data.
(TIF)

S24 Fig Natural metabolic pathways of C1–C6 carbon sources. (**A**) Natural metabolic pathways of methanol. RuMP, ribulose monophosphate pathway; XuMP, xylulose monophosphate pathway; EMP, Embden–Meyerhoff–Parnas pathway. (**B**) Natural metabolic pathways of ethylene glycol. BHAC, β-hydroxyaspartate cycle. (**C**) Natural metabolic pathways of glycerol. (**D**) Natural metabolic pathways of erythritol. PPP, pentose phosphate pathway. (**E**) Natural metabolic pathways of D-xylose. (**F**) Natural metabolic pathways of D-glucose.
(TIF)

S1 Table Activity of PKs from different species on different substrates. (XLSX)

S2 Table Energies and energy corrections for the forming process of DHEThDP from DHA. (XLSX)

S3 Table Energies and energy corrections for the forming process of DHEThDP from D-EUS. (XLSX)

S4 Table Energies and energy corrections for the forming process of DHEThDP from L-EUS. (XLSX)

S5 Table Enzyme kinetic parameters. (DOCX)

S6 Table The standard Gibbs free energy change (ΔG’) of the BbPK for various sugars. (XLSX)

S7 Table Overall reactions and properties of various carbon metabolism. (DOCX)

S8 Table List of plasmids. (DOCX)

S9 Table List of synthesized genes and their optimized sequences. (XLSX)

S1 Data Excel spreadsheet containing the underlying numerical data for Figs 2B, 3B, 4C–F, S13, S14, S15, S16, S17, S21, and S23. (XLSX)

S1 Raw Images The raw image for S19 Fig. (TIF)

## Abbreviations

AcP: acetyl-phosphate
APK: artificial phosphoketolase
D-EUS: D-erythrulose
DHA: dihydroxyacetone
DHAP: dihydroxyacetone phosphate
EI: electron ionization
F6P: fructose-6-phosphate
FLS: formolase
GALD: glycolaldehyde
IPTG: isopropyl-β-D-thiogalactopyranoside
PK: phosphoketolase
PTA: phosphate acetyltransferase
TFA: aqueous trifluoroacetic acid
ThDP: thiamin diphosphate
Xu5P: xylulose-5-phosphate

## References

[1] Garrett RH, Grisham CM. Biochemistry. 2nd ed. Saunders College Publishing.

[2] Nielsen J, Keasling JD. Engineering cellular metabolism. Cell. 2016;164(6):1185–1197. doi: 10.1016/j.cell.2016.02.004 26967285

[3] Xu K, Yin N, Peng M, Stamatiades EG, Shyu A, Li P, et al. Glycolysis fuels phosphoinositide 3-kinase signaling to bolster T cell immunity. Science. 2021;371(6527):405–410. doi: 10.1126/science.abb2683 33479154 PMC8380312

[4] Bollong MJ, Lee G, Coukos JS, Yun H, Zambaldo C, Chang JW, et al. A metabolite-derived protein modification integrates glycolysis with KEAP1–NRF2 signalling. Nature. 2018;562(7728):600–604. doi: 10.1038/s41586-018-0622-0 30323285 PMC6444936

[5] Sukonina V, Ma H, Zhang W, Bartesaghi S, Subhash S, Heglind M, et al. FOXK1 and FOXK2 regulate aerobic glycolysis. Nature. 2019;566(7743):279–283. doi: 10.1038/s41586-019-0900-5 30700909

[6] Bailey JE. Toward a science of metabolic engineering. Science. 1991;252(5013):1668–1675. doi: 10.1126/science.2047876 2047876

[7] Atsumi S, Hanai T, Liao JC. Non-fermentative pathways for synthesis of branched-chain higher alcohols as biofuels. Nature. 2008;451(7174):86–89. doi: 10.1038/nature06450 18172501

[8] Meadows AL, Hawkins KM, Tsegaye Y, Antipov E, Kim Y, Raetz L, et al. Rewriting yeast central carbon metabolism for industrial isoprenoid production. Nature. 2016;537(7622):694–697. doi: 10.1038/nature19769 27654918

[9] Opgenorth PH, Korman TP, Bowie JU. A synthetic biochemistry module for production of bio-based chemicals from glucose. Nat Chem Biol. 2016;12(6):393–395. doi: 10.1038/nchembio.2062 27065234

[10] Wang M, Chen B, Fang Y, Tan T. Cofactor engineering for more efficient production of chemicals and biofuels. Biotechnol Adv. 2017;35(8):1032–1039. doi: 10.1016/j.biotechadv.2017.09.008 28939499

[11] Noor E, Eden E, Milo R, Alon U. Central carbon metabolism as a minimal biochemical walk between precursors for biomass and energy. Mol Cell. 2010;39(5):809–820. doi: 10.1016/j.molcel.2010.08.031 20832731

[12] Bar-Even A, Flamholz A, Noor E, Milo R. Rethinking glycolysis: on the biochemical logic of metabolic pathways. Nat Chem Biol. 2012;8(6):509–517. doi: 10.1038/nchembio.971 22596202

[13] Bogorad IW, Lin T-S, Liao JC. Synthetic non-oxidative glycolysis enables complete carbon conservation. Nature. 2013;502(7473):693–697. doi: 10.1038/nature12575 24077099

[14] Hellgren J, Godina A, Nielsen J, Siewers V. Promiscuous phosphoketolase and metabolic rewiring enables novel non-oxidative glycolysis in yeast for high-yield production of acetyl-CoA derived products. Metab Eng. 2020;62:150–160. doi: 10.1016/j.ymben.2020.09.003 32911054

[15] Suzuki R, Katayama T, Kim B-J, Wakagi T, Shoun H, Ashida H, et al. Crystal structures of phosphoketolase: thiamine diphosphate-dependent dehydration mechanism. J Biol Chem. 2010;285(44):34279–34287. doi: 10.1074/jbc.M110.156281 20739284 PMC2962526

[16] Tittmann K. Sweet siblings with different faces: the mechanisms of FBP and F6P aldolase, transaldolase, transketolase and phosphoketolase revisited in light of recent structural data. Bioorg Chem. 2014;57:263–280. doi: 10.1016/j.bioorg.2014.09.001 25267444

[17] Zhang J, Liu Y. Computational studies on the catalytic mechanism of phosphoketolase. Comput Theor Chem. 2013;1025:1–7.

[18] Granström TB, Takata G, Tokuda M, Izumori K. Izumoring: a novel and complete strategy for bioproduction of rare sugars. J Biosci Bioeng. 2004;97(2):89–94. doi: 10.1016/S1389-1723(04)70173-5 16233597

[19] Izumori K. Izumoring: a strategy for bioproduction of all hexoses. J Biotechnol. 2006;124(4):717–722. doi: 10.1016/j.jbiotec.2006.04.016 16716430

[20] Lu X, Liu Y, Yang Y, Wang S, Wang Q, Wang X, et al. Constructing a synthetic pathway for acetyl-coenzyme A from one-carbon through enzyme design. Nat Commun. 2019;10(1):1–10. 30914637 10.1038/s41467-019-09095-z PMC6435721

[21] Suzuki R, Kim B-J, Shibata T, Iwamoto Y, Katayama T, Ashida H, et al. Overexpression, crystallization and preliminary X-ray analysis of xylulose-5-phosphate/fructose-6-phosphate phosphoketolase from Bifidobacterium breve. Acta Crystallogr Sect F Struct Biol Cryst Commun. 2010;66(8):941–943. doi: 10.1107/S1744309110023845 20693675 PMC2917298

[22] Toteva MM, Silvaggi NR, Allen KN, Richard JP. Binding energy and catalysis by D-xylose isomerase: kinetic, product, and X-ray crystallographic analysis of enzyme-catalyzed isomerization of (R)-glyceraldehyde. Biochemistry. 2011;50(46):10170–10181. doi: 10.1021/bi201378c 21995300 PMC3217161

[23] Leang K, Takada G, Fukai Y, Morimoto K, Granström TB, Izumori K. Novel reactions of L-rhamnose isomerase from Pseudomonas stutzeri and its relation with D-xylose isomerase via substrate specificity. Biochim Biophys Acta. 2004;1674(1):68–77. doi: 10.1016/j.bbagen.2004.06.003 15342115

[24] Siegel JB, Smith AL, Poust S, Wargacki AJ, Bar-Even A, Louw C, et al. Computational protein design enables a novel one-carbon assimilation pathway. Proc Natl Acad Sci U S A. 2015;112(12):3704–3709. doi: 10.1073/pnas.1500545112 25775555 PMC4378393

[25] de Jong BW, Shi S, Siewers V, Nielsen J. Improved production of fatty acid ethyl esters in Saccharomyces cerevisiae through up-regulation of the ethanol degradation pathway and expression of the heterologous phosphoketolase pathway. Microb Cell Fact. 2014;13(1):1–10. doi: 10.1186/1475-2859-13-39 24618091 PMC3995654

[26] Henard CA, Smith HK, Guarnieri MT. Phosphoketolase overexpression increases biomass and lipid yield from methane in an obligate methanotrophic biocatalyst. Metab Eng. 2017;41:152–158. doi: 10.1016/j.ymben.2017.03.007 28377275

[27] Chwa JW, Kim WJ, Sim SJ, Um Y, Woo HM. Engineering of a modular and synthetic phosphoketolase pathway for photosynthetic production of acetone from CO_2_ in Synechococcus elongatus PCC 7942 under light and aerobic condition. Plant Biotechnol J. 2016;14(8):1768–1776. 26879003 10.1111/pbi.12536 PMC5021146

[28] Jumper J, Evans R, Pritzel A, Green T, Figurnov M, Ronneberger O, et al. Highly accurate protein structure prediction with AlphaFold. Nature. 2021;596(7873):583–589. doi: 10.1038/s41586-021-03819-2 34265844 PMC8371605

[29] Delano WL. Pymol: An open-source molecular graphics tool. 2002;40(1):82–92.

[30] Frisch MJ, Trucks GW, Schlegel HB, Scuseria GE, Robb MA, Cheeseman JR, et al. Gaussian 09, Gaussian, Inc., Wallingford, CT, 2009.

[31] Marenich AV, Cramer CJ, Truhlar DG. Universal solvation model based on solute electron density and on a continuum model of the solvent defined by the bulk dielectric constant and atomic surface tensions. J Phys Chem B. 2009;113(18):6378–6396. doi: 10.1021/jp810292n 19366259

[32] Planas F, McLeish MJ, Himo F. Computational Study of Enantioselective Carboligation Catalyzed by Benzoylformate Decarboxylase. ACS Catalysis. 2019;9(6):5657–5667.

[33] Sheng X, Himo F. Computational Study of Pictet–Spenglerase Strictosidine Synthase: Reaction Mechanism and Origins of Enantioselectivity of Natural and Non-Natural Substrates. ACS Catalysis. 2020;10(22):13630–40.

[34] McNutt AT, Francoeur P, Aggarwal R, Masuda T, Meli R, Ragoza M, et al. GNINA 1.0: molecular docking with deep learning. J Cheminform. 2021;13(1):43. doi: 10.1186/s13321-021-00522-2 34108002 PMC8191141

[35] Eastman P, Swails J, Chodera JD, McGibbon RT, Zhao Y, Beauchamp KA, et al. OpenMM 7: Rapid development of high performance algorithms for molecular dynamics. PLoS Comput Biol. 2017;13(7):e1005659. doi: 10.1371/journal.pcbi.1005659 28746339 PMC5549999

[36] Literature W, Case T, Babin V, Berryman J, Betz RM, Cai Q, et al. AMBER 14, University of California, San Francisco, 2014.

[37] Wheeler TJ, Eddy SR. nhmmer: DNA homology search with profile HMMs. Bioinformatics. 2013;29(19):2487–2489. doi: 10.1093/bioinformatics/btt403 23842809 PMC3777106

[38] Hug LA, Baker BJ, Anantharaman K, Brown CT, Probst AJ, Castelle CJ, et al. A new view of the tree of life. Nat Microbiol. 2016;1(5):1–6. doi: 10.1038/nmicrobiol.2016.48 27572647

