# Supplementary figures and images for: Construction of an artificial phosphoketolase pathway that efficiently catabolizes multiple carbon sources to acetyl-CoA
Source: PLoS Biol. 2023 Sep 21;21(9):e3002285. doi: 10.1371/journal.pbio.3002285 (PMC10547157; doi:10.1371/journal.pbio.3002285)

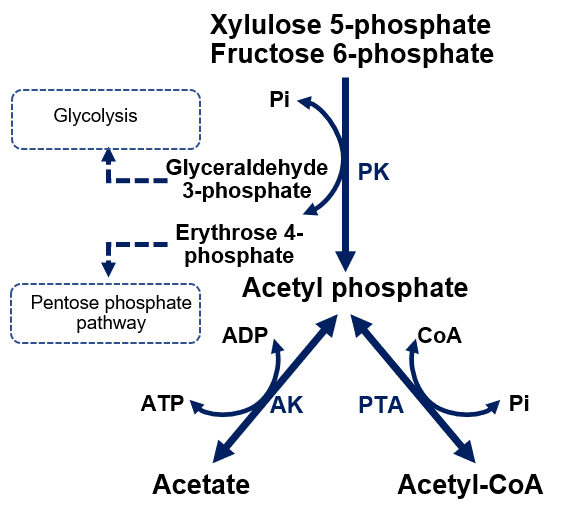

Supplement: S1 Fig — Fructose-6-phosphate (F6P) and xylulose-5-phosphate (Xu5P) are converted into D-erythorse-4-phosphate/glyceraldehyde-3-phosphate (E4P/G3P) and acetyl-phosphate (AcP), which not only can be converted into acetyl-CoA by phosphate acetyltransferase (PTA), but also can be converted to ATP and acetate by acetate kinase (AK). (TIF) [file pbio.3002285.s001.tif]

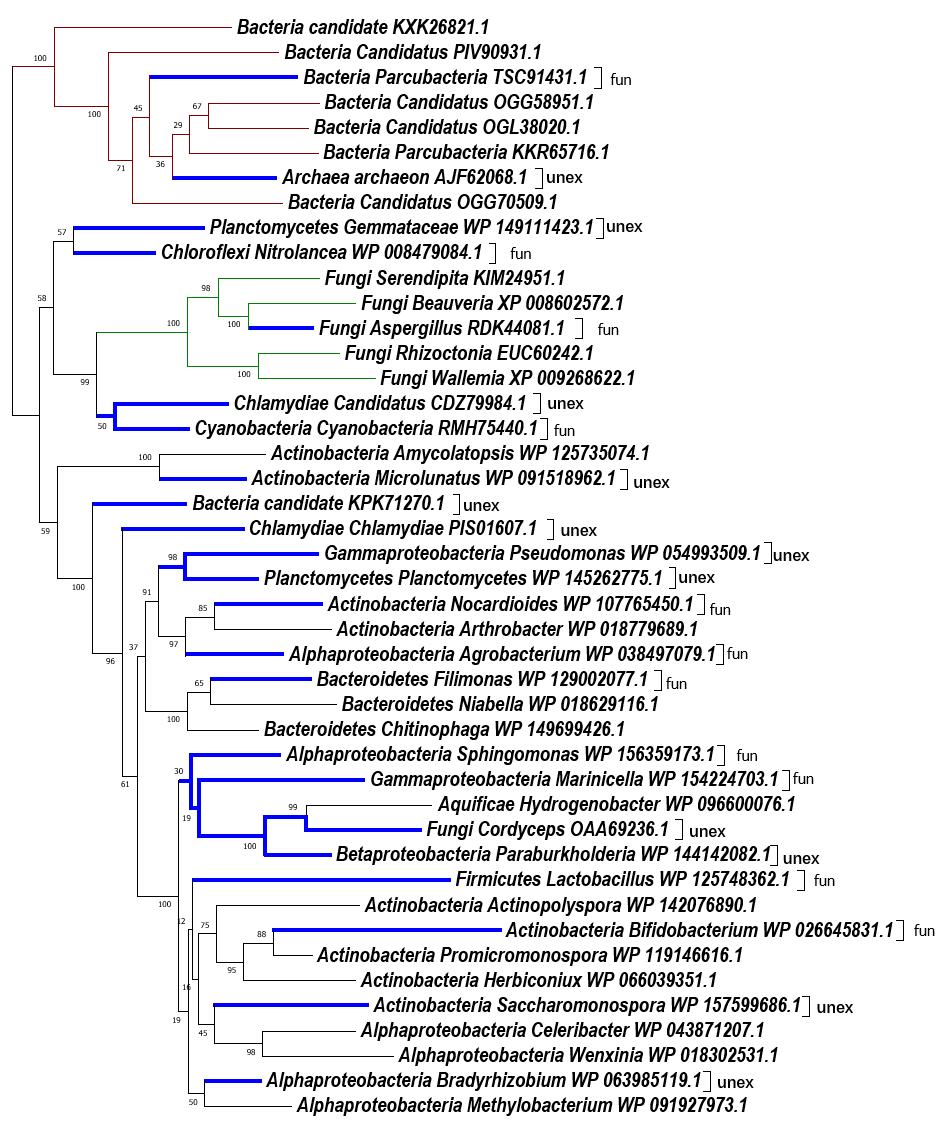

Supplement: S2 Fig — “]” indicates that the species has been selected to test. “unex” indicates that the protein is not expressed correctly in E. coli. “fun” indicates that PKs have activities on F6P or Xu5P. (TIF) [file pbio.3002285.s002.tif]

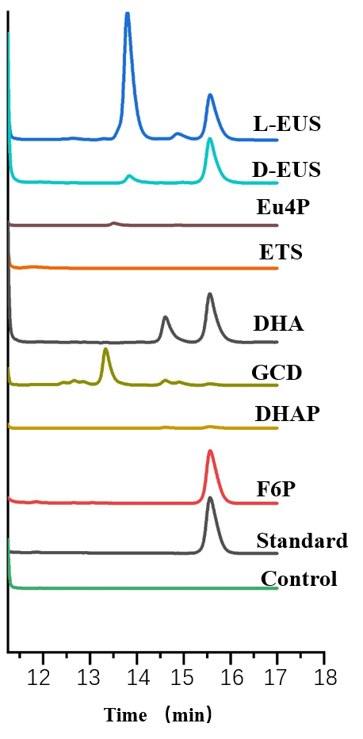

Supplement: S3 Fig — The product acetyl phosphate was converted to acetic acid, which was detected by HPLC. The reaction system without PK was used as the control. L-EUS, L-erythrulose; D-EUS, D-erythrulose; Eu4P, D-erythrulose-4-phosphate; ETS, D-erythrose; DHA, dihydroxyacetone; GCD, D-glyceraldehyde; DHAP, dihydroxyacetone phosphate; F6P, D-fructose-6-phosphate. (TIF) [file pbio.3002285.s003.tif]

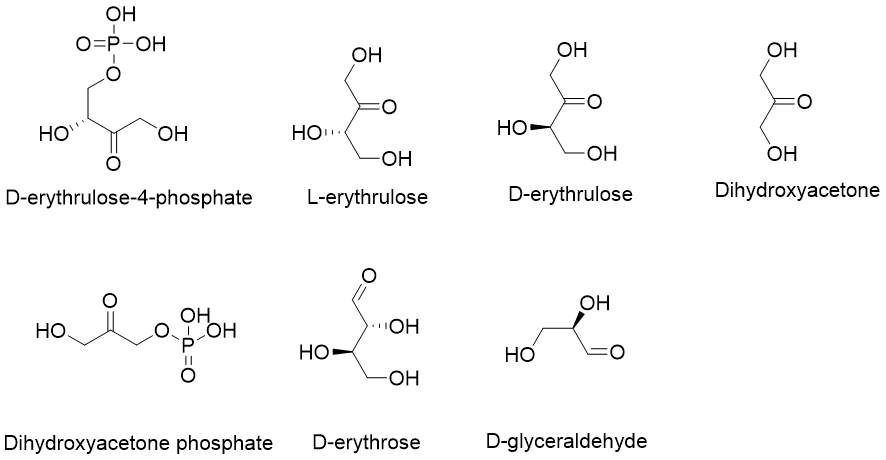

Supplement: S4 Fig — (TIF) [file pbio.3002285.s004.tif]

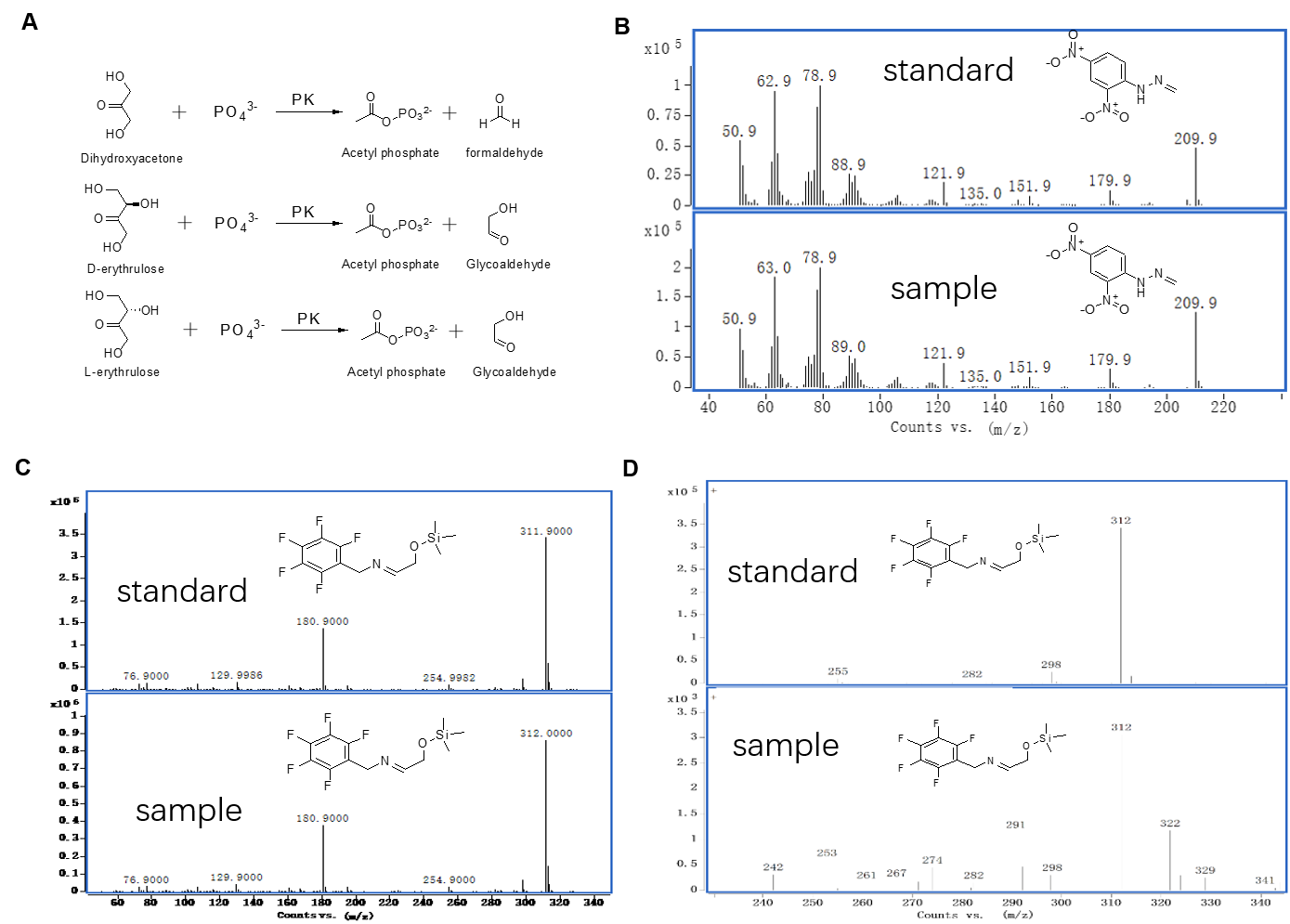

Supplement: S5 Fig — (A) Schematic illustration of the reaction of dihydroxyacetone, D-erythrulose, and L-erythrulose catalyzed by PK. (B) Formaldehyde was detected by GC-MS in PK-catalyzed dihydroxyacetone reaction system. (C) Glycoaldehyde was detected by GC-MS in PK-catalyzed D-erythrulose reaction system. (D) Glycoaldehyde was detected by GC-MS in PK-catalyzed L-erythrulose reaction system. Sample derivatization methods, see Materials and methods. (TIF) [file pbio.3002285.s005.tif]

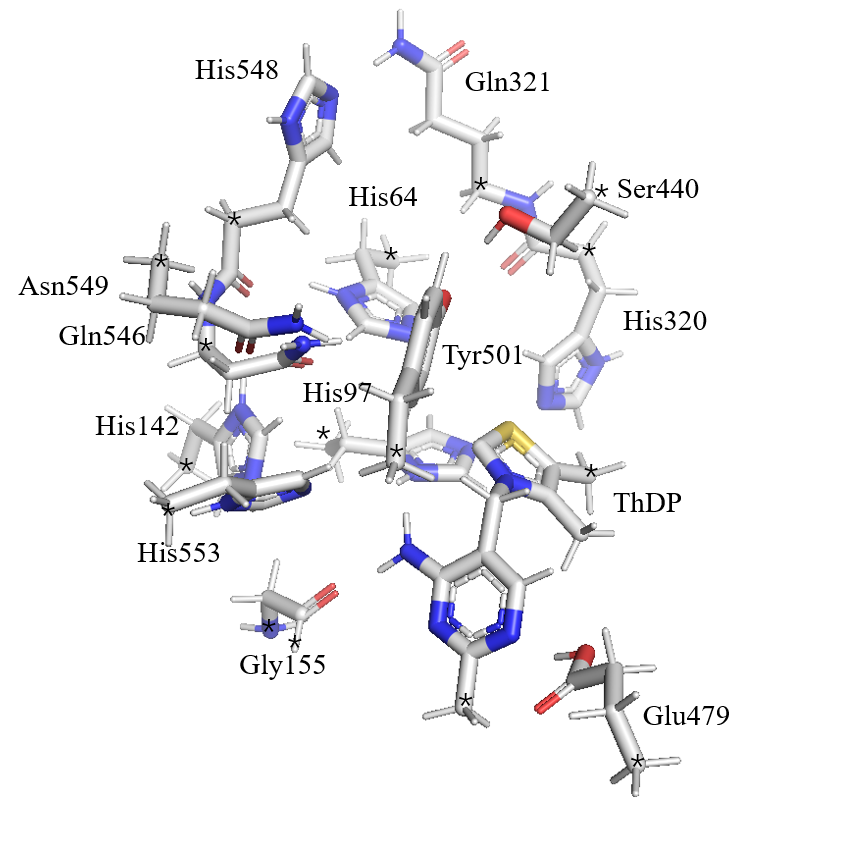

Supplement: S6 Fig — The model contains 218 atoms with a total charge of +1, including the side chains of His64, His553, Glu479, Tyr501, Gln321, Ser440, His142, Gly155, His320, Asn549, Gln546, His548, His97, and the cofactor ThDP. The fixed atoms are labeled by asterisks. (TIF) [file pbio.3002285.s006.tif]

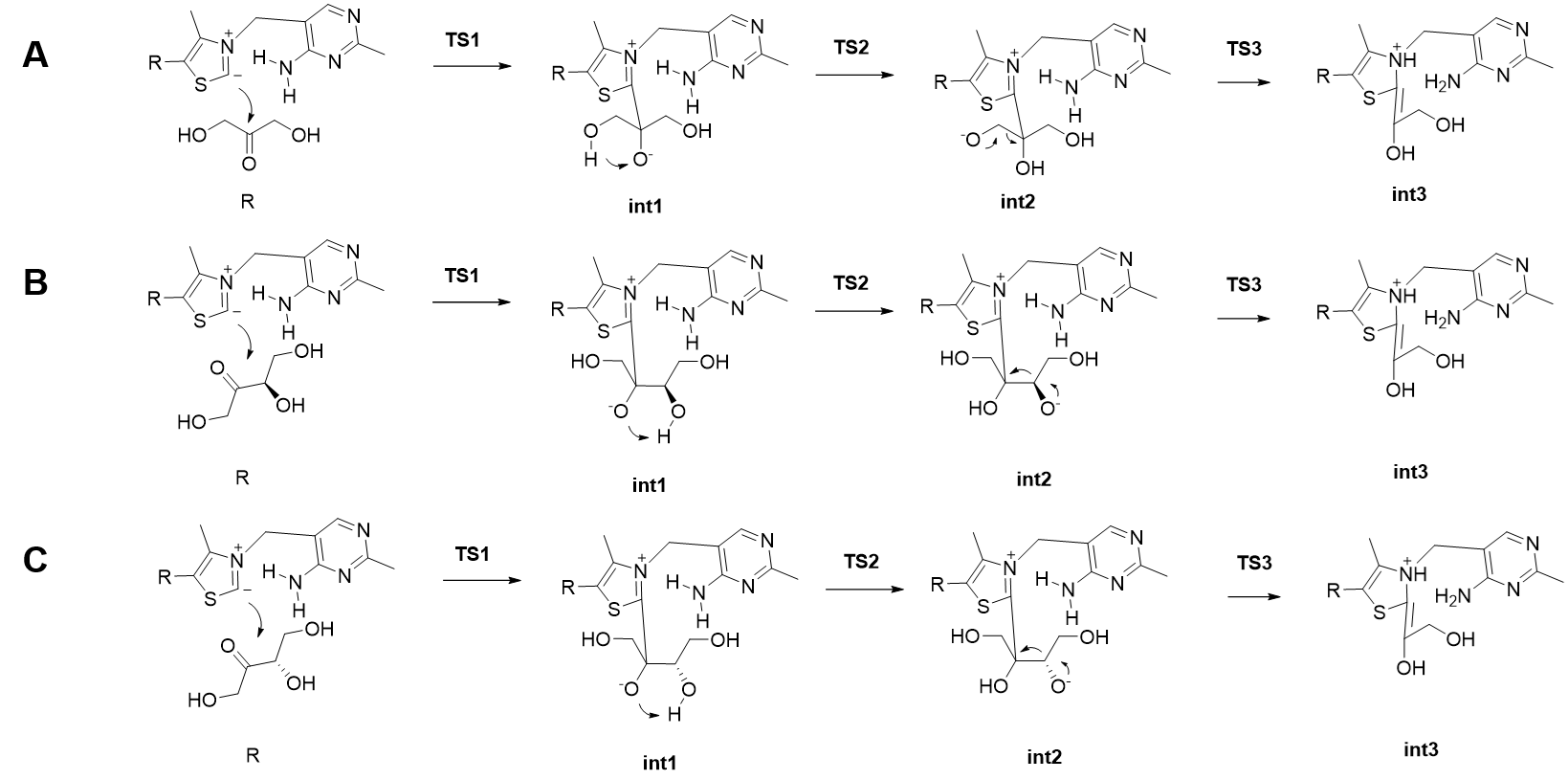

Supplement: S7 Fig — (A) The forming process of 2-α, β-dihydroxyethylidene-ThDP (DHEThDP) (int3) from 1,3-dihydroxyacetone. (B) The forming process of DHEThDP from D-erythrulose. (C) The forming process of DHEThDP from L-erythrulose. Upon binding of the substrate to ThDP, the first step is a C−C bond formation that leads to an alkoxide tetrahedral intermediate. Next, an intramolecular proton transfer takes place from the hydroxyl group in C3 to the alkoxide. The last step is a C−C bond cleavage to form the DHEThDP. R, reactant; int1, intermediate 1; TS1, transition state 1. (TIF) [file pbio.3002285.s007.tif]

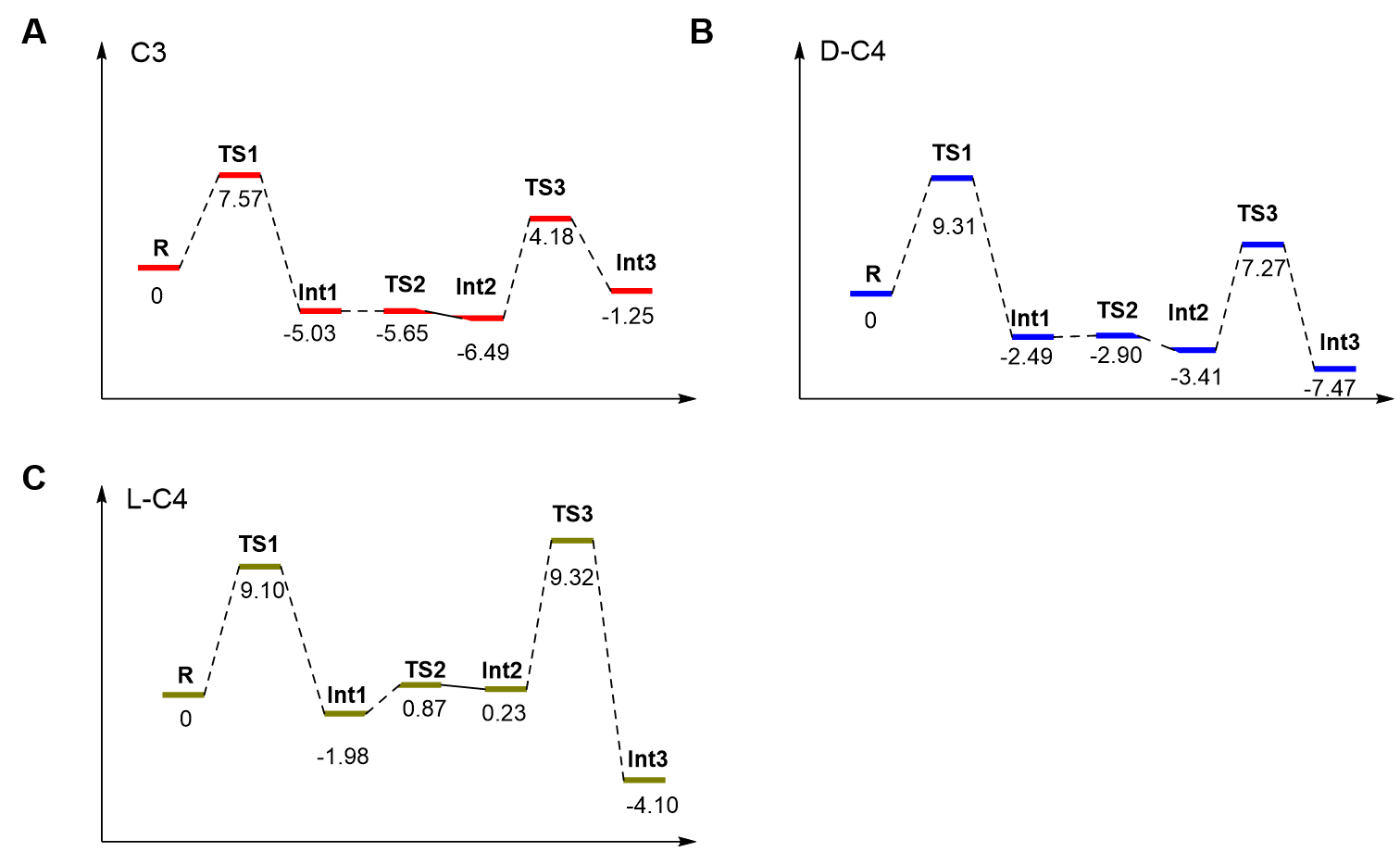

Supplement: S8 Fig — (A) The energy profiles for 1,3-dihydroxyacetone. (B) The energy profiles for D-erythrulose. (C) The energy profiles for L-erythrulose. Energies are given in kilocalories per mole. Note: After adding the large basis set, solvation, and zero-point energy corrections, the energies of TS2 for 1, 3-dihydroxyacetone and D-erythrulose were calculated to be lower than those of int1. Therefore, intramolecular proton transfer of 1,3-dihydroxyacetone and D-erythrulose can be assumed to be barrierless or to occur with very low barriers. (TIF) [file pbio.3002285.s008.tif]

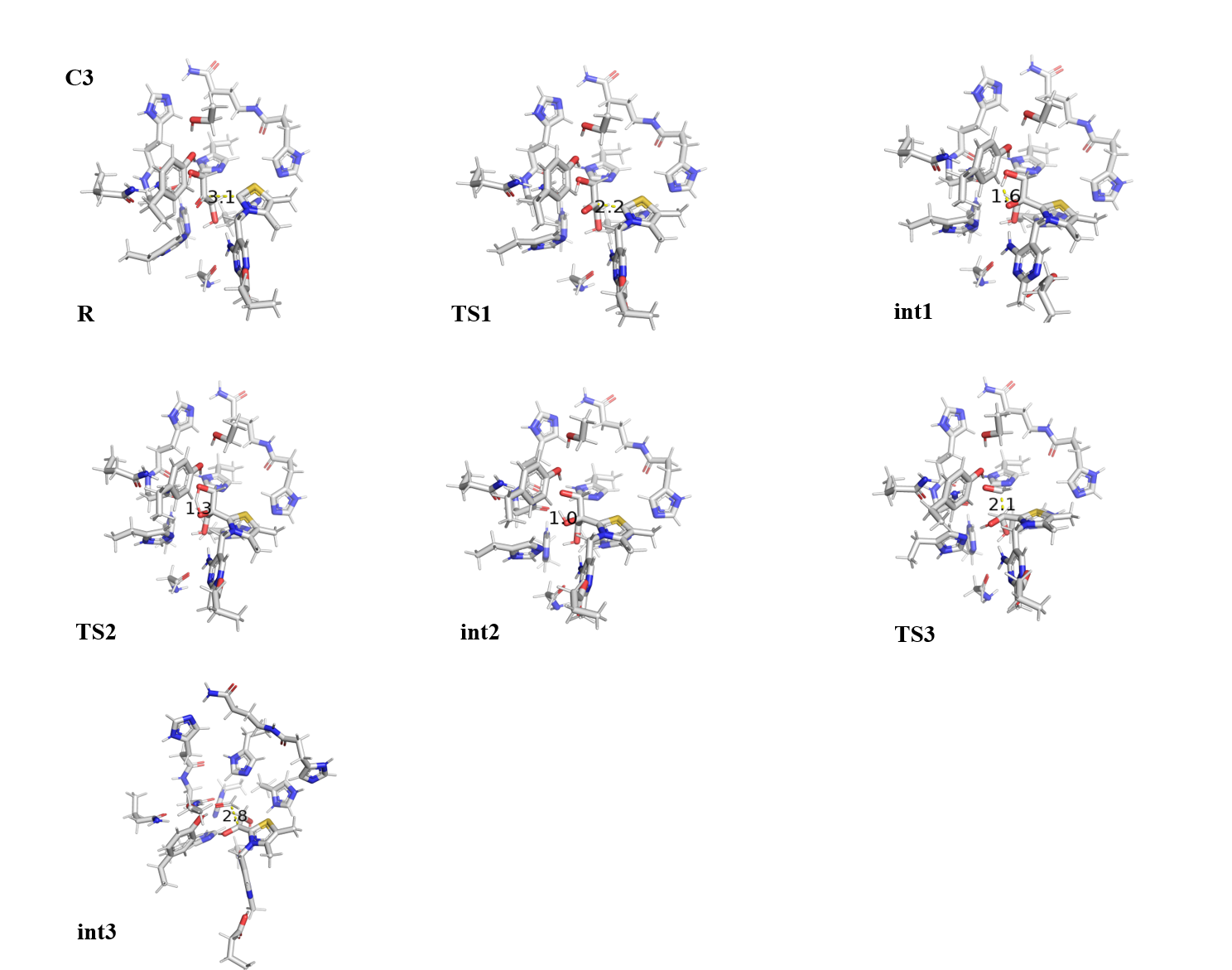

Supplement: S9 Fig — The key bond distances change is shown in the figure. Selected distances are given in Å. The distances between C2 of ThDP and carbonyl C of substrate changes from 3.1 Å in Reactant (R) to 2.2 Å in transition state 1 (TS1). The distance between O and H changes from 1.6 Å in intermediate 1 (int1) to 1.3 Å in TS2. The distance of C2 and C3 of substrate changes from 2.1 Å in TS3 to 2.8 Å in int3. (TIF) [file pbio.3002285.s009.tif]

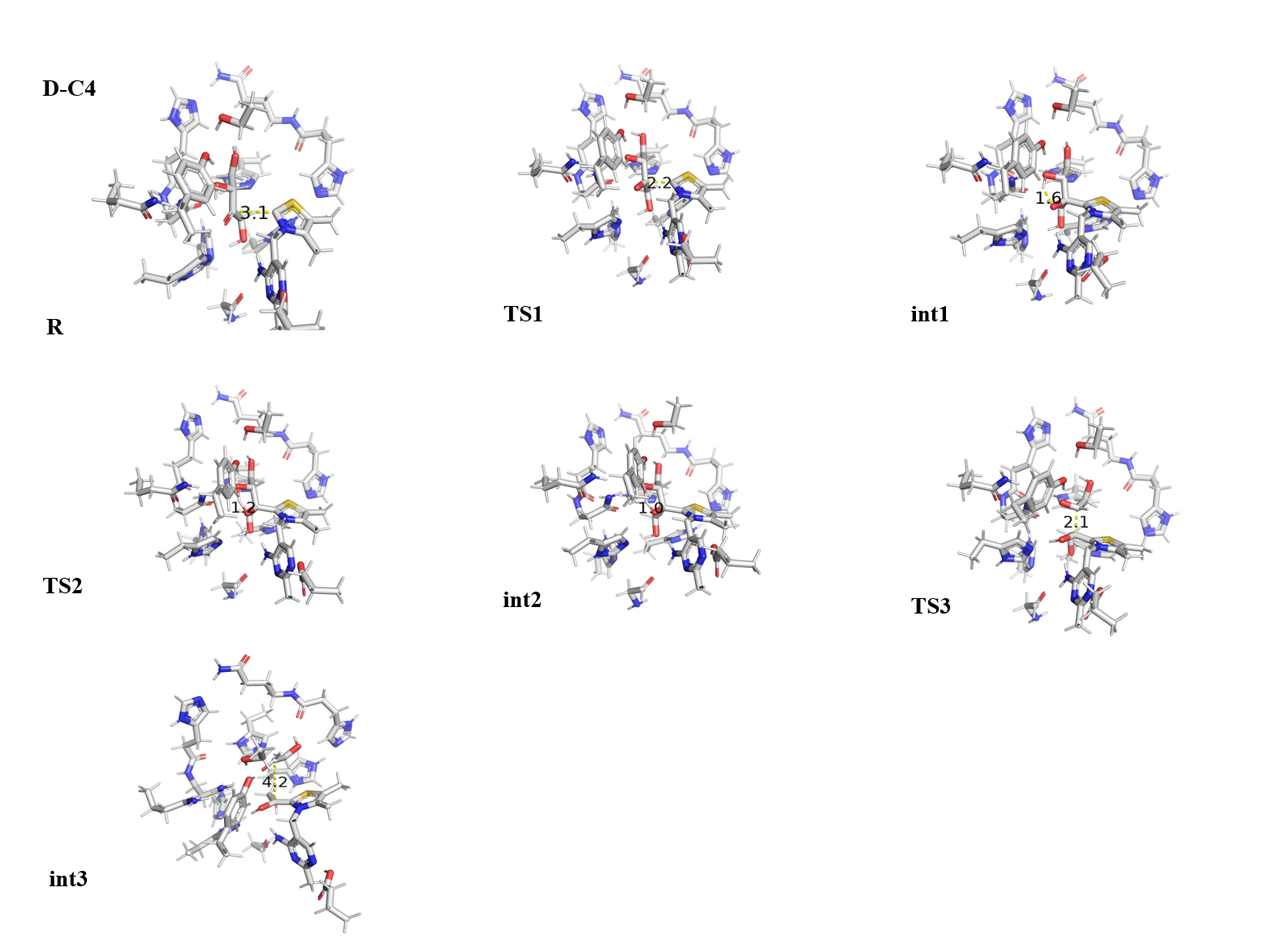

Supplement: S10 Fig — The key bond distances change is shown in the figure. Selected distances are given in Å. The distances between C2 of ThDP and carbonyl C of substrate changes from 3.1 Å in Reactant (R) to 2.2 Å in transition state 1 (TS1). The distance between O and H changes from 1.6 Å in intermediate 1 (int1) to 1.2 Å in TS2. The distance of C2 and C3 of substrate changes from 2.1 Å in TS3 to 4.2 Å in int3. (TIF) [file pbio.3002285.s010.tif]

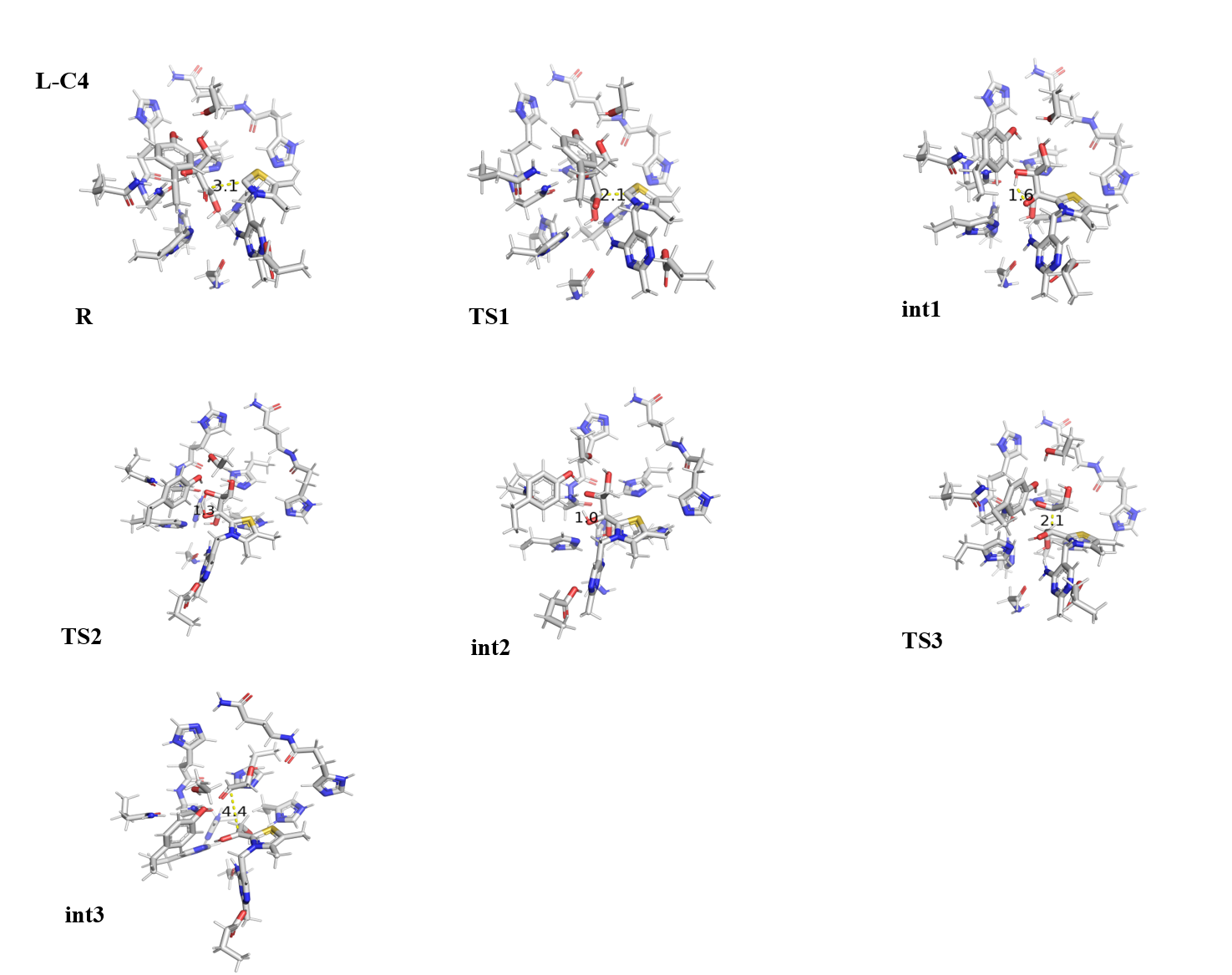

Supplement: S11 Fig — The key bond distances change is shown in the figure. Selected distances are given in Å. The distances between C2 of ThDP and carbonyl C of substrate changes from 3.1 Å in Reactant (R) to 2.1 Å in transition state 1 (TS1). The distance between O and H changes from 1.6 Å in intermediate 1 (int1) to 1.3 Å in TS2. The distance of C2 and C3 of substrate changes from 2.1 Å in TS3 to 4.4 Å in int3. (TIF) [file pbio.3002285.s011.tif]

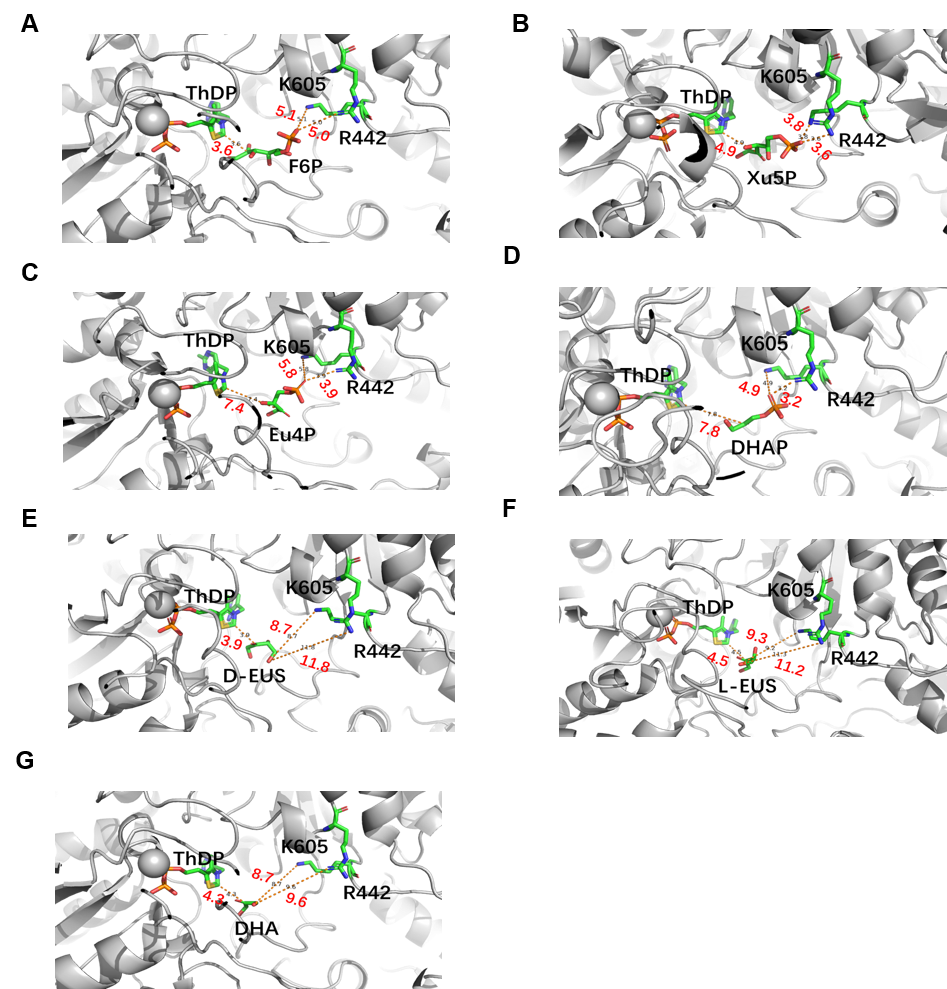

Supplement: S12 Fig — The PK was shown in cartoon and colored gray. The ThDP, ligands, and 2 key residues R442 and K605 were shown in stick. The C, N, O, P, and S atoms were colored green, blue, red, orange, and yellow, respectively. The distance between the substrates and ThDP and the distances between the phosphate moiety of substrates and the key basic residues were shown as dashed lines. Selected distances are given in Å. (TIF) [file pbio.3002285.s012.tif]

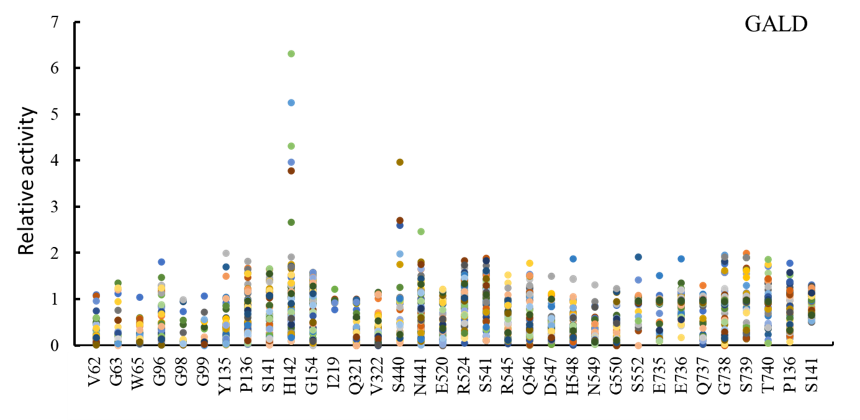

Supplement: S13 Fig — The x-axis labels represent the selected location in the BbPK. The y-axis labels represent the relative catalytic activities of the different mutants. Relative activity was defined as the ratio of the reduction of substrate for mutants to that of the wild type. The raw data was listed in S1 Data. (TIF) [file pbio.3002285.s013.tif]

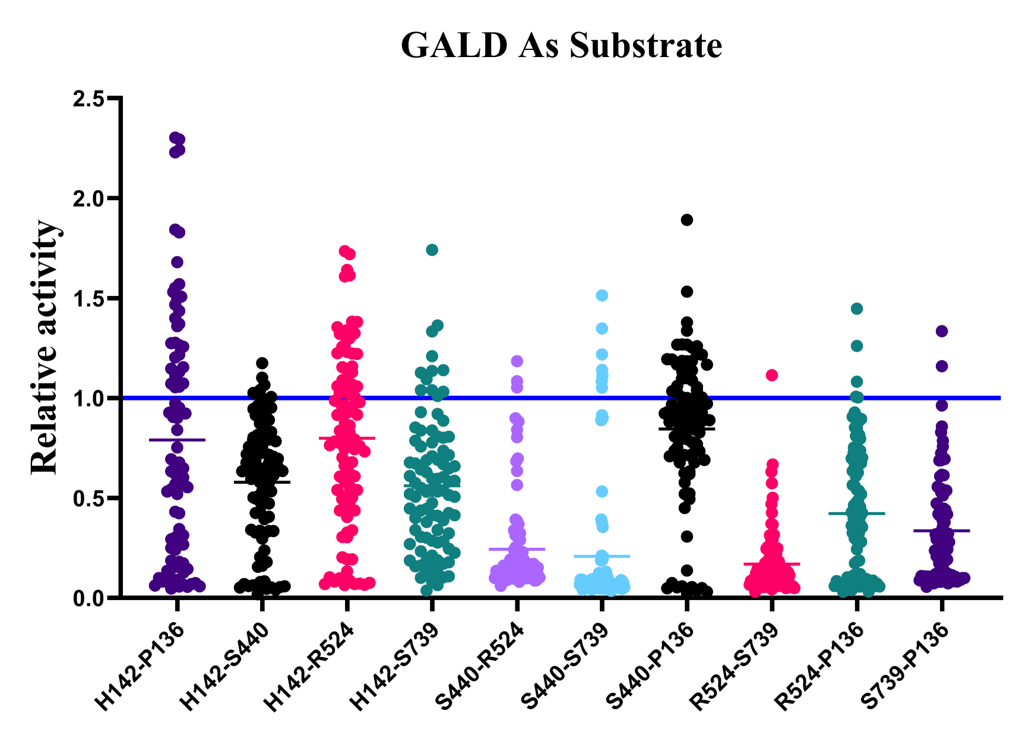

Supplement: S14 Fig — The x-axis labels represent the location combinations in the BbPK. The y-axis labels represent the relative catalytic activities of the different mutants. Relative activity was defined as the ratio of the reduction of substrate for mutants to that of the wild type. The raw data was listed in S1 Data. (TIF) [file pbio.3002285.s014.tif]

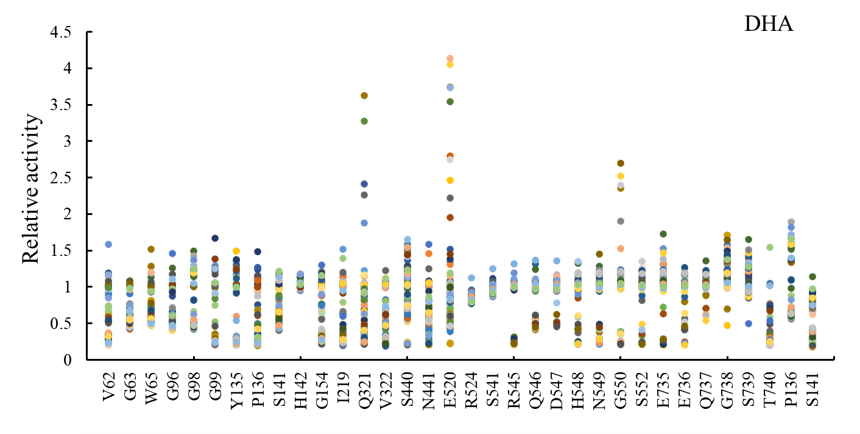

Supplement: S15 Fig — The x-axis labels represent the selected location in the BbPK. The y-axis labels represent the relative catalytic activities of the different mutants. Relative activity was defined as the ratio of the titer of formaldehyde for the mutants to that of the wild type. The raw data was listed in S1 Data. (TIF) [file pbio.3002285.s015.tif]

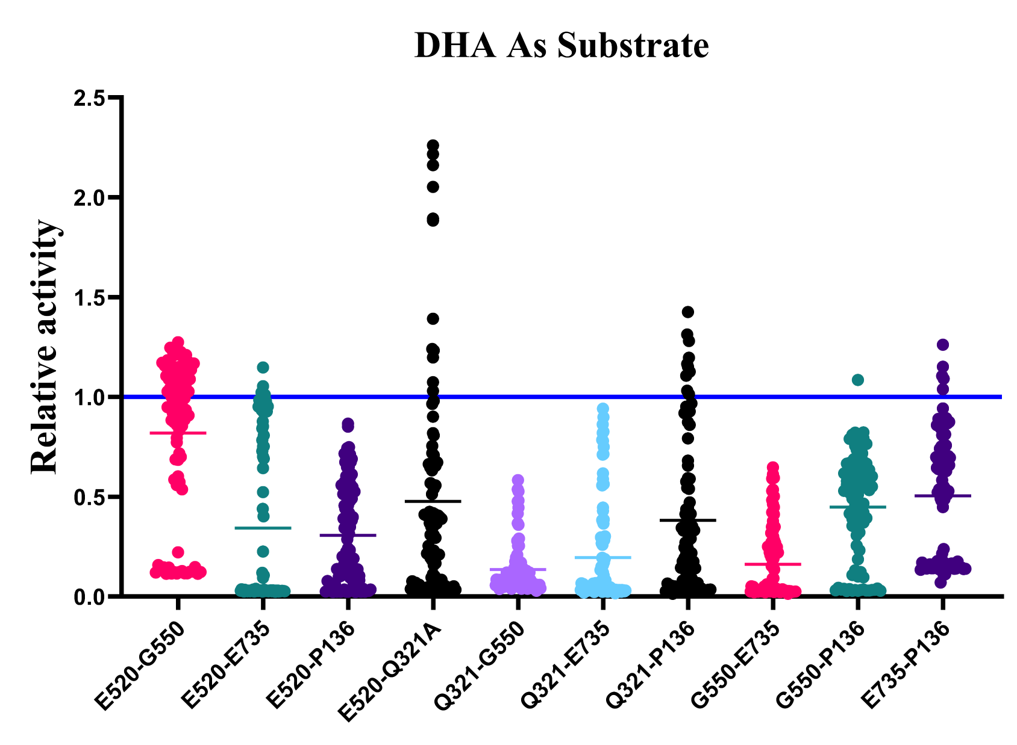

Supplement: S16 Fig — The x-axis labels represent the location combinations in the BbPK. The y-axis labels represent the relative catalytic activities of the different mutants. Relative activity was defined as the ratio of the titer of formaldehyde for the mutants to that of the wild type. The raw data was listed in S1 Data. (TIF) [file pbio.3002285.s016.tif]

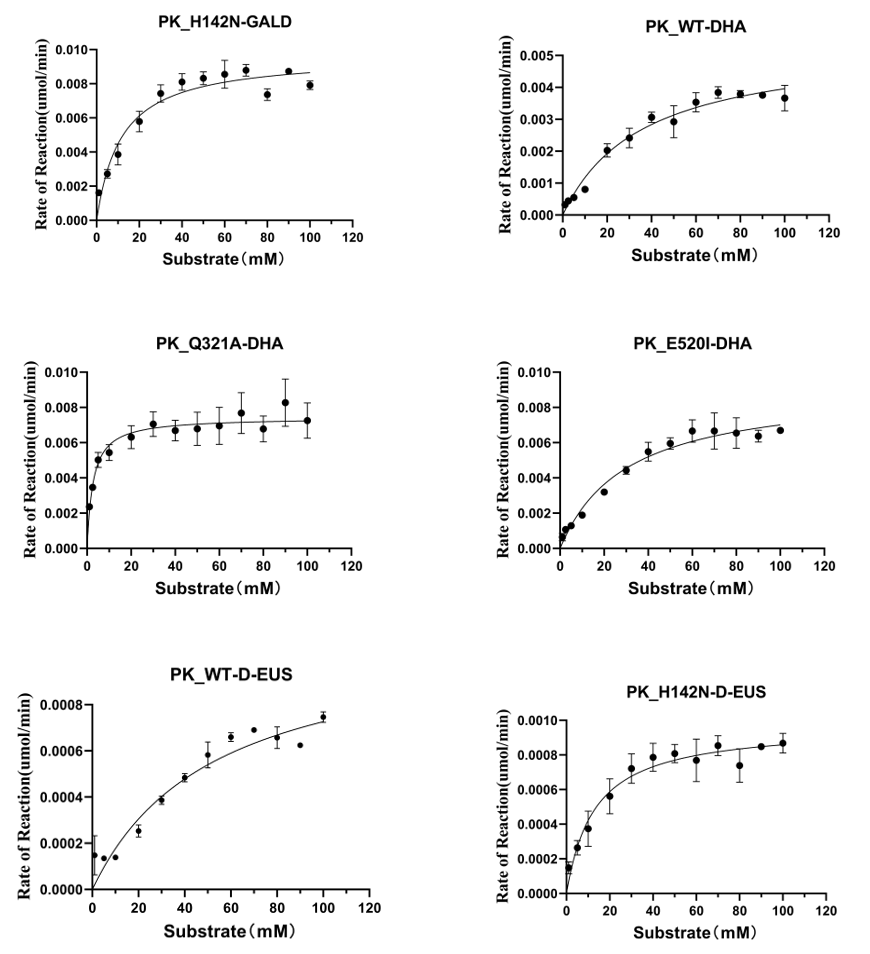

Supplement: S17 Fig — Enzyme kinetics were determined with 0.25 mg mL−1 enzyme. The concentration of substrate ranged from 1 to 110 mM. Error bars represent SD (standard deviation), n = 3. The raw data was listed in S1 Data. (TIF) [file pbio.3002285.s017.tif]

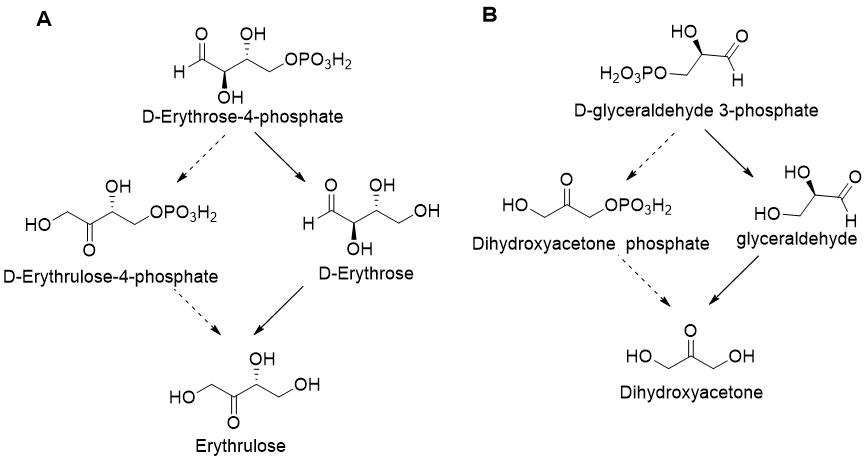

Supplement: S18 Fig — (A) The 2 conversion pathways of D-erythrulose (D-EUS) from D-erythrose-4-phosphate (E4P). (B) The 2 conversion pathways of dihydroxyacetone (DHA) from D-glyceraldehyde-3-phosphate (G3P). Dashed arrows represent the pathway that is first isomerized and then dephosphorylated. Solid arrows represent the pathway that is first dephosphorylated and then isomerized. (TIF) [file pbio.3002285.s018.tif]

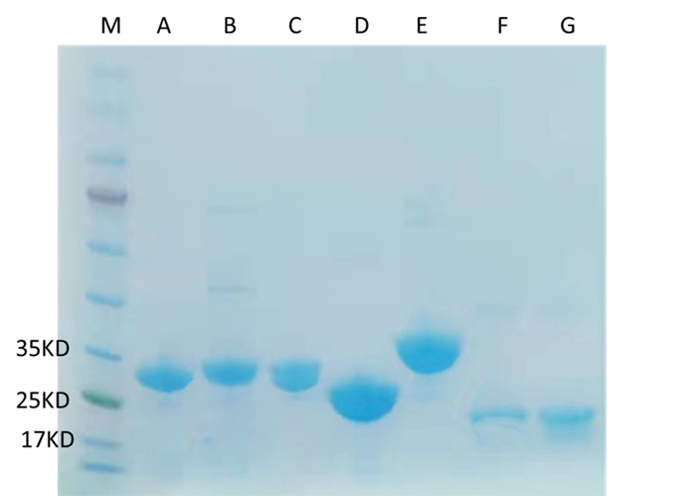

Supplement: S19 Fig — (A), pfHAD; (B), EcHAD; (C), NbIMP; (D), TmHAD; (E), CpHAD; (F), XiHAD; (G), CgHAD. The detailed information of phosphatases, see S8 and S9 Tables. (TIF) [file pbio.3002285.s019.tif]

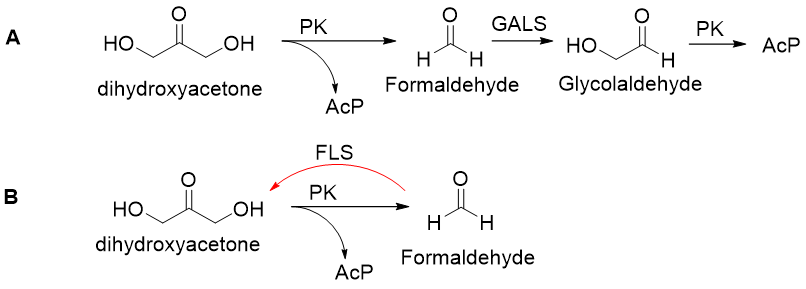

Supplement: S20 Fig — (A) Formaldehyde is converted to glycolaldehyde by glycolaldehyde synthase (GALS). Glycolaldehyde is then converted to AcP by PK. (B) Formaldehyde is converted to dihydroxyacetone by formolase (FLS). Dihydroxyacetone is then converted to AcP by PK. (TIF) [file pbio.3002285.s020.tif]

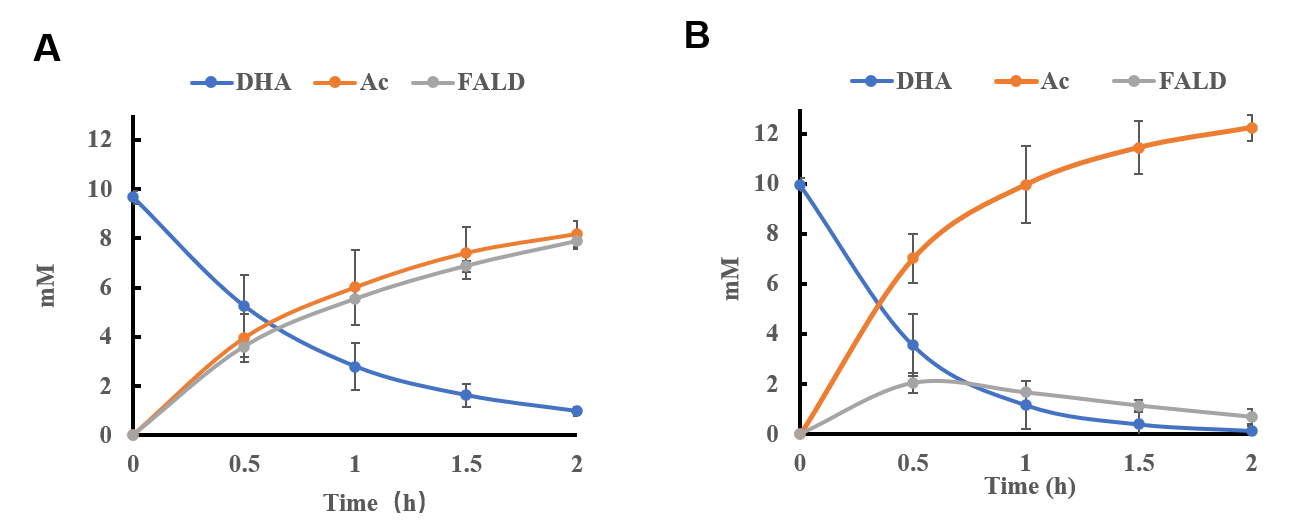

Supplement: S21 Fig — (A) The conversion of DHA to AcP using only BbPK in vitro. (B) Formolase (FLS) was used to recycle formaldehyde. The red curve represents the change of acetic acid concentration. The blue curve represents the change of DHA concentration. The gray curve represents the change of formaldehyde concentration. Acetic acid was detected by HPLC. Error bars represent SD (standard deviation), n = 3. The raw data was listed in S1 Data. (TIF) [file pbio.3002285.s021.tif]

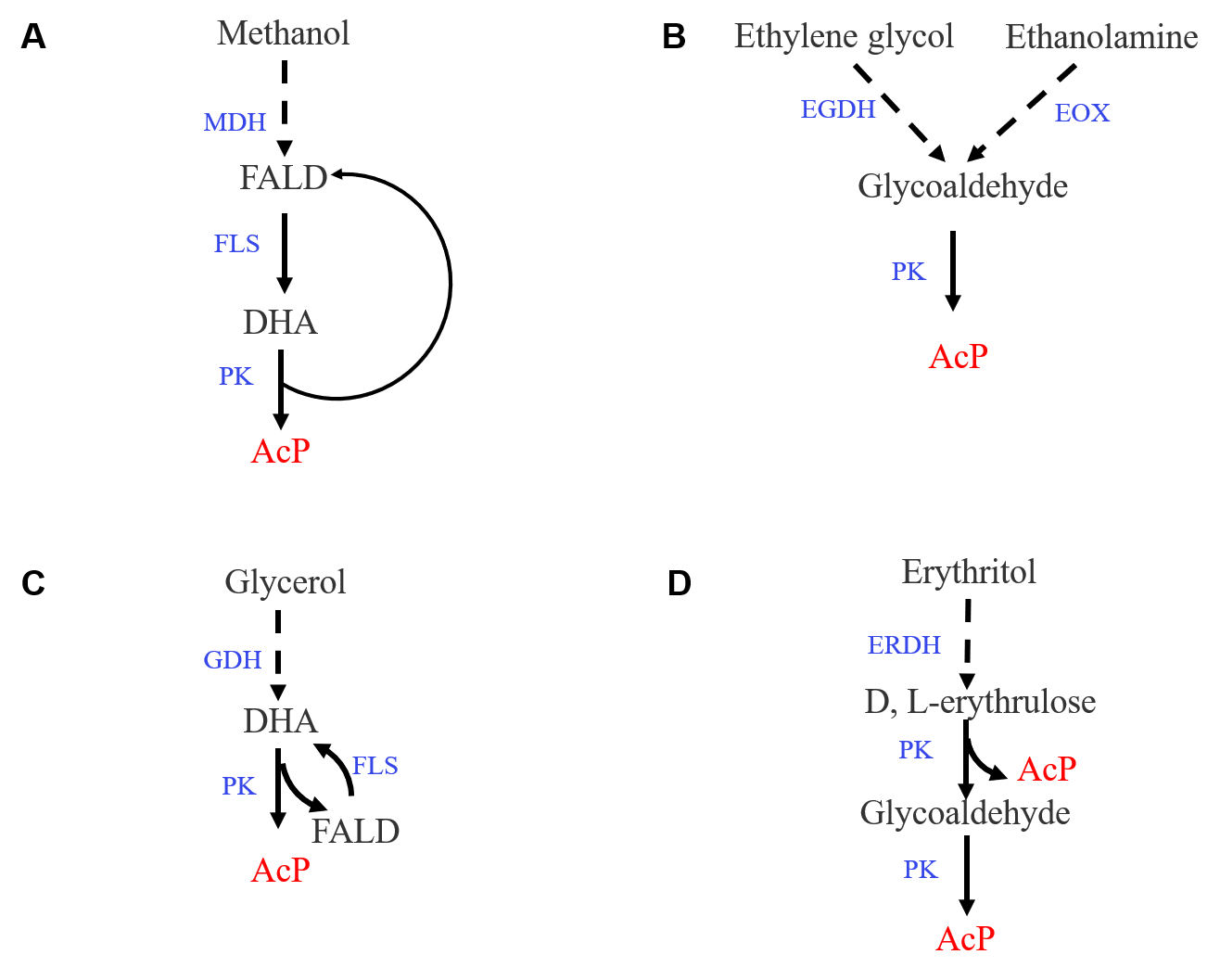

Supplement: S22 Fig — (A) Methanol is converted to DHA and then converted to AcP via APK pathway. MDH, methanol dehydrogenase. (B) Ethylene glycol or ethanolamine is converted to glycoaldehyde and then converted to AcP via APK pathway. EGDH, ethylene glycol dehydrogenase; EOX, ethanolamine oxidase. (C) Glycerol is converted to DHA and then converted to AcP via APK pathway. GDH, glycerol dehydrogenase. (D) Erythritol is converted to erythrulose and then converted to AcP via APK pathway. ERDH, erythritol dehydrogenase. Dashed arrows represent the process that is not tested. (TIF) [file pbio.3002285.s022.tif]

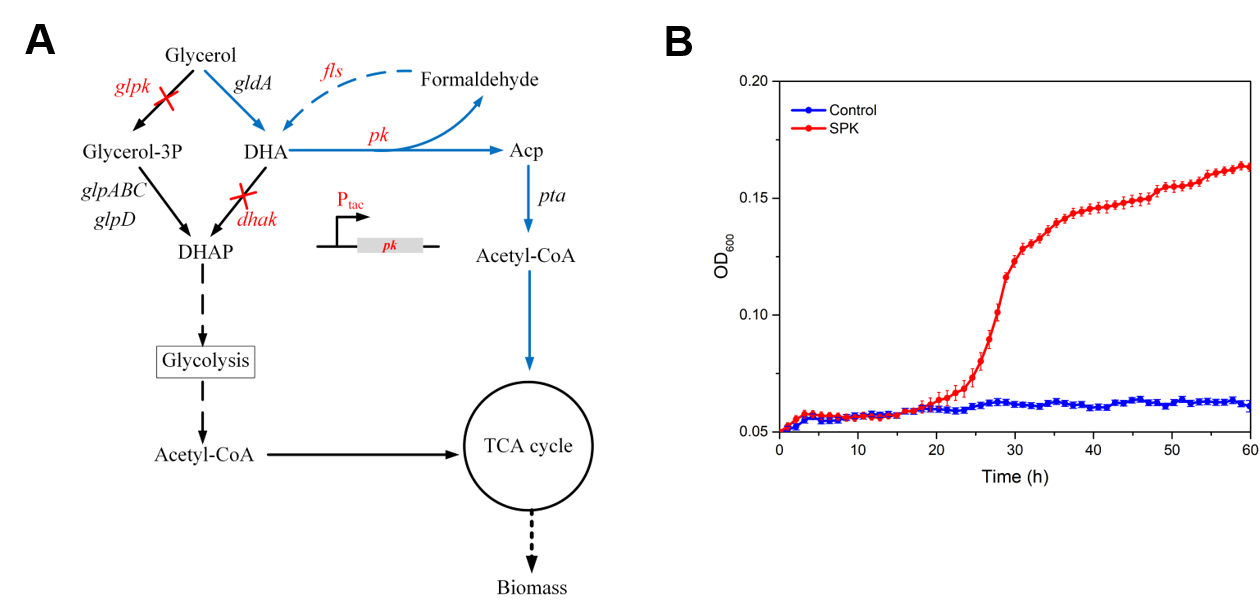

Supplement: S23 Fig — (A) Schematic representation of the natural glycerol metabolic pathways and APK pathway. Red crosses represent gene knockout. Blue arrows represent APK pathway. The promoter Ptac was used to overexpress pk in the plasmid. Metabolite abbreviation: DHA, dihydroxyacetone; DHAP, dihydroxyacetone phosphate; AcP, acetyl phosphate. Genes involved: glpk, glycerol kinase; dhak, dihydroxyacetone kinase. (B) Growth curve in glycerol minimal medium. The control was MG1655ΔglpkΔdhak. The strain PK (SPK) was MG1655ΔglpkΔdhak harboring plasmid pBD-PK. Plasmid pBD-PK was constructed for the expression of pk under the control of the Ptac promoter. Error bars represent SD (standard deviation), n = 3. The raw data was listed in S1 Data. (TIF) [file pbio.3002285.s023.tif]

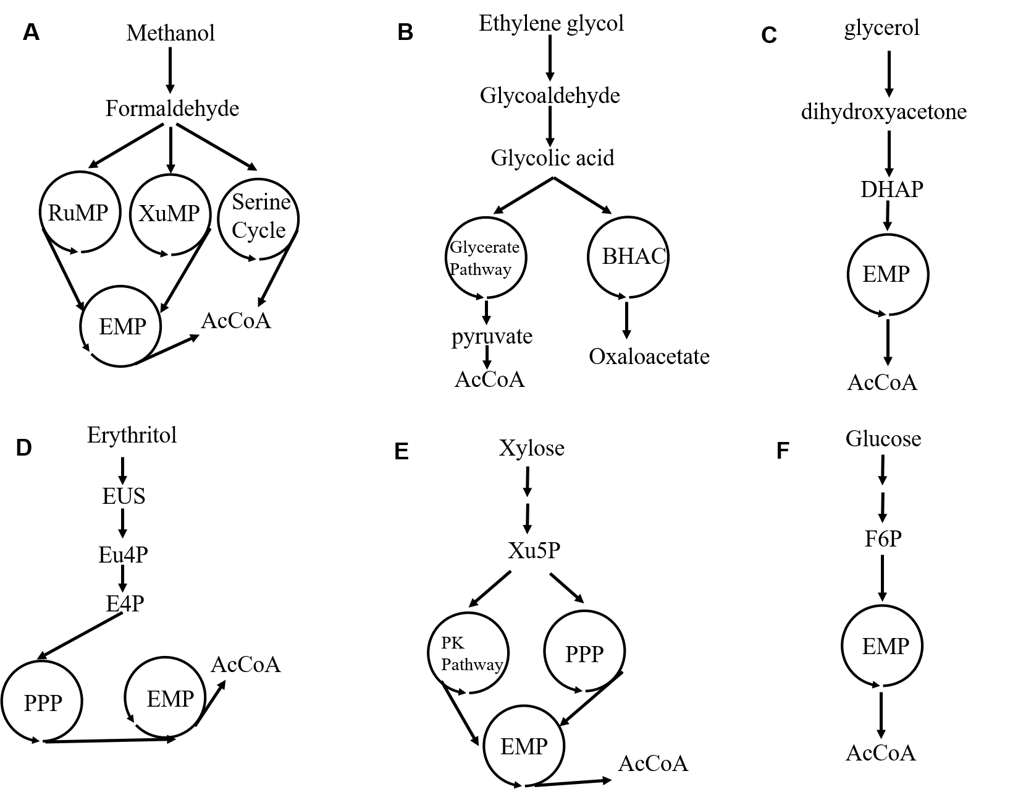

Supplement: S24 Fig — (A) Natural metabolic pathways of methanol. RuMP, ribulose monophosphate pathway; XuMP, xylulose monophosphate pathway; EMP, Embden–Meyerhoff–Parnas pathway. (B) Natural metabolic pathways of ethylene glycol. BHAC, β-hydroxyaspartate cycle. (C) Natural metabolic pathways of glycerol. (D) Natural metabolic pathways of erythritol. PPP, pentose phosphate pathway. (E) Natural metabolic pathways of D-xylose. (F) Natural metabolic pathways of D-glucose. (TIF) [file pbio.3002285.s024.tif]

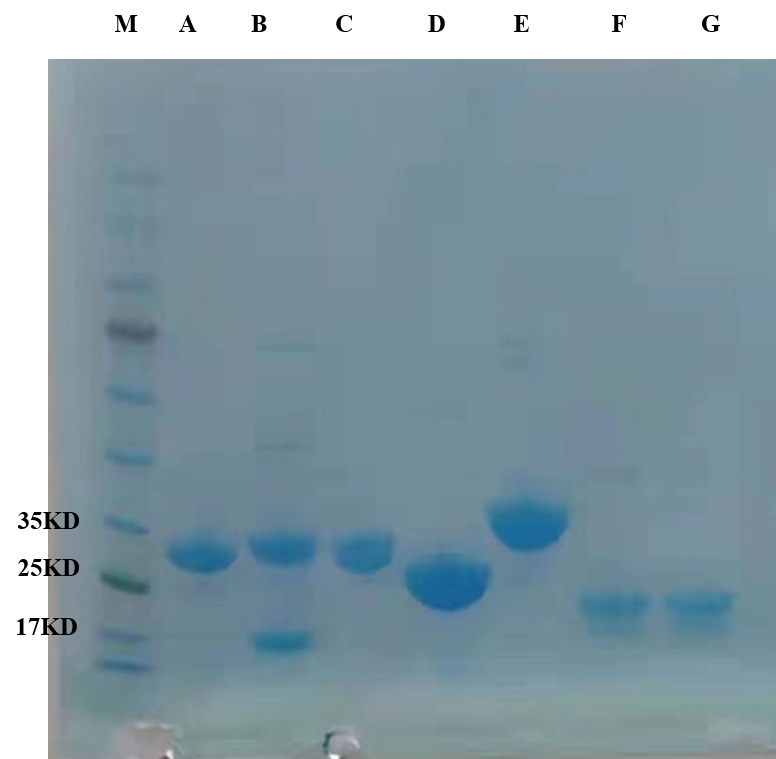

Supplement: S1 Raw Images — (TIF) [file pbio.3002285.s035.tif]
