# Supplementary material for: Construction of an artificial phosphoketolase pathway that efficiently catabolizes multiple carbon sources to acetyl-CoA
Source: PLoS Biol. 2023 Sep 21;21(9):e3002285. doi: 10.1371/journal.pbio.3002285 (PMC10547157; doi:10.1371/journal.pbio.3002285)
Supplement: S5 Table — (DOCX) [file pbio.3002285.s029.docx]

**Table S5. Enzyme kinetic parameters**

| Variants | Substrate | *k*_cat_ (s^-1^) | *K*_m_ (mM) | *k*_cat_/*K*_m_  (s^-1^· M ^-1^) | Source |
| --- | --- | --- | --- | --- | --- |
| FLS | FALD | 0.15 | 34.14 | 4.40 | [1] |
| GALS | FALD | 1.58 | 170 | 9.30 | [2] |
| BbPK | GALD | 0.16 | 51 | 3.10 | [2] |
| PK-H142N | GALD | 0.31 | 11.55 | 26.38 | This study |
| BbPK | DHA | 0.17 | 35.88 | 3.80 | This study |
| PK-E520I | DHA | 0.29 | 30.23 | 8.70 | This study |
| PK-Q321A | DHA | 0.23 | 12.47 | 18.82 | This study |
| BbPK | D-EUS | 0.035 | 52.93 | 0.66 | This study |
| PK-H142N | D-EUS | 0.031 | 13.04 | 2.35 | This study |
| BbPK | F6P | 520 | 10 | 52000 | [3] |
| BbPK | Xu5P | 2700 | 45 | 60000 | [3] |

**References**

1. Siegel JB, Smith AL, Poust S, Wargacki AJ, Bar-Even A, Louw C, et al. Computational protein design enables a novel one-carbon assimilation pathway. Proceedings of the National Academy of Sciences of the United States of America. 2015;112(12):3704-9.

2. Lu X, Liu Y, Yang Y, Wang S, Wang Q, Wang X, et al. Constructing a synthetic pathway for acetyl-coenzyme A from one-carbon through enzyme design. Nature communications. 2019;10(1):1378.

3. Meile L, Rohr LM, Geissmann TA, Herensperger M, Teuber M. Characterization of the D-xylulose 5-phosphate/D-fructose 6-phosphate phosphoketolase gene (xfp) from Bifidobacterium lactis. Journal of bacteriology. 2001;183(9):2929-36.
