# Supplementary material for: Construction of an artificial phosphoketolase pathway that efficiently catabolizes multiple carbon sources to acetyl-CoA
Source: PLoS Biol. 2023 Sep 21;21(9):e3002285. doi: 10.1371/journal.pbio.3002285 (PMC10547157; doi:10.1371/journal.pbio.3002285)
Supplement: S7 Table — (DOCX) [file pbio.3002285.s031.docx]

**Table S7. Overall reactions and properties of various carbon metabolism**

| Pathway | Product for glucose | Net ATP  for glucose  and DHA | Net redox | Types of metabolic carbon sources | Enzymes | Kinetic trap | Carbon yield  % |
| --- | --- | --- | --- | --- | --- | --- | --- |
| MAG | 3 acetate | 1 and 1.5 | No | C1 C2, C3, C4, C5, C6, | 6 | No | 100 |
| EMP | 2 lactate | 1 and 1 | No | C3, C6 | 11 | No | 67 |
| ED | 2 lactate | 1 and 1 | No | C3, C6 | 11 | No | 67 |
| PPP | 6 CO_2_ | 0 | 6 | C3, C5, C6 | 11 | No | 0 |
| GLYP | - | - | - | C2, C3 | - | No | <67 |
| BHAC | - | - | - | C2 | - | No | <67 |
| RuMP | - | - | - | C1 | - | No | <67 |
| XuMP | - | - | - | C1 | - | No | <67 |
| WL | - | - | - | C1 | - | No | 100 |
| PKP | lactate + ethanol + CO_2_ | 1 | No | C6, C5, C3 | 15 | No | - |
| B-S | 1.5 acetate + lactate | 2.5 | No | C6, C5, C3 | 14 | No | - |
| NOG[1] | 3 acetate | 2 | No | C6, C5, C3 | 11 | Yes | 100 |
| GATHCYC | 3 acetate | 2 | No | C6 | 8 | No | 100 |

Note: ED, Entner–Doudoroff pathway; GLYP, glycerate pathway; BHAC, β-hydroxyaspartate cycle; WL, Wood−Ljungdahl pathway; PKP, phosphoketolase pathway; B-S, bifid shunt; GATHCYC, Glycolysis AlTernative High Carbon Yield Cycle.

**References**

1. Bogorad IW, Lin TS, Liao JC. Synthetic non-oxidative glycolysis enables complete carbon conservation. Nature. 2013;502(7473):693-7.
