# Supplementary material for: Construction of an artificial phosphoketolase pathway that efficiently catabolizes multiple carbon sources to acetyl-CoA
Source: PLoS Biol. 2023 Sep 21;21(9):e3002285. doi: 10.1371/journal.pbio.3002285 (PMC10547157; doi:10.1371/journal.pbio.3002285)
Supplement: S8 Table — (DOCX) [file pbio.3002285.s032.docx]

**Table S8. List of plasmids.**

| **Plasmids** | **Short discription** | **Source** |
| --- | --- | --- |
| pET28a-RpiB | pET28a based vector, carrying *rpiB* gene from *Brucella abortus*; Kan^r^ | This study |
| pET28a-EcHAD | pET28a based vector, carrying *yidA* gene from *Escherichia coli* MG1655; Kanr | This study |
| pET28a-NbIMP | pET28a based vector, carrying *impase* gene from *Nocardia brasiliensis*; Kan^r^ | This study |
| pET28a-TmHAD | pET28a based vector, carrying *had* gene from *Thermotoga,* Kan^r^ | This study |
| pET28a-XlHAD | pET28a based vector, carrying *had* gene from *Xenopus laevis*; Kan^r^ | This study |
| pET28a-pfHAD | pET28a based vector, carrying *had* gene from *Plasmodium falciparum*; Kan^r^ | This study |
| pET28a-nagD | pET28a based vector, carrying *had* gene from *Corynebacterium*; Kan^r^ | This study |
| pET28a-CpHAD | pET28a based vector, carrying *had* gene from *Candida parapsilosis*; Kan^r^ | This study |
| pET28a-pk-1 | pET28a based vector, carrying *pk* gene from Lactobacillus baoqingensis; Kan^r^ | This study |
| pET28a-pk-2 | pET28a based vector, carrying *pk* gene from Aspergillus niger; Kan^r^ | This study |
| pET28a-pk-3 | pET28a based vector, carrying *pk* gene from *Pseudomonas congelans*; Kan^r^ | This study |
| pET28a-pk-4 | pET28a based vector, carrying *pk* gene from *Bifidobacterium adolescentis;* Kanr | This study |
| pET28a-pk-5 | pET28a based vector, carrying *pk* gene from *Agrobacterium tumefaciens complex*; Kan^r^ | This study |
| pET28a-pk-6 | pET28a based vector, carrying *pk* gene from *Nitrolancea hollandica*; Kan^r^ | This study |
| pET28a-pk-7 | pET28a based vector, carrying *pk* gene from *Sphingomonas sp*; Kan^r^ | This study |
| pET28a-pk-8 | pET28a based vector, carrying *pk* gene from *Parcubacteria group bacterium*; Kan^r^ |  |
| pET28a-pk-9 | pET28a based vector, carrying *pk* gene from; *Cyanobacteria bacterium J007* Kan^r^ |  |
| pET28a-pk-10 | pET28a based vector, carrying *pk* gene from; *Filimonas effuse* *J007* Kan^r^ |  |
| pET28a-pk-11 | pET28a based vector, carrying *pk* gene from; *Nocardioides terrigena* Kan^r^ |  |
| pET28a-fls | pET28a based vector, carrying *fls* gene from *Methanosarcina thermophila*; Kane^r^ | This study |
| pET28a-PsLrhi | pET28a based vector, carrying *PsLrhi* gene from *Pseudomonas stutzeri*; Kane^r^ | [1] |
| pET28a-gals | pET28a based vector, carrying *gals* gene; Kane^r^ | [2] |
| pET28a-ack | pET28a based vector, carrying *ackA* gene from *E. coli* Mg1655; Kan^r^ | [3] |
| pET28a-TIM | pET28a based vector, carrying *tpiA* gene from *E. coli* Mg1655; Kanr | This study |
| pACYC-DuetI-pk-4- *Pslrhi* | pET28a based vector, carrying *pk4* and *Pslrhi* ; Cm^r^ | This study |
| pACYC-DuetI-pk4-fls | pET28a based vector, carrying *pk4* and *fls*gene from *Myceliophthora thermophila*; Cm^r^ | This study |
| pTargetT-ldhA | pMB1 *ldhA* sgRNA-pMB，Spec^r^ | This study |
| pTargetT-adhE | pMB1 *adhE* sgRNA-pMB1，Spec^r^ | This study |
| pTargetT-pflB | pMB1 *pflB* sgRNA-pMB1，Spec^r^ | This study |
| pTargetT-fuco | pMB1 *fucO* sgRNA-pMB1，Spec^r^ | This study |
| pTargetT-frdBC | pMB1 *frdBC* sgRNA-pMB1，Spec^r^ | This study |
| pTargetT-yqhD | pMB1 *yqhD* sgRNA-pMB1，Spec^r^ | This study |
| pTargetT-aldA | pMB1 *aldA* sgRNA-pMB1，Spec^r^ | This study |
| pTargetT-patI | pMB1 *ptaI* sgRNA-pMB1，Spec^r^ | This study |
| pTargetT-acs | pMB1 *acs* sgRNA-pMB1，Spec^r^ | This study |
| pTargetT-zwf | pMB1 *zwf* sgRNA-pMB1，Spec^r^ | This study |
| pTargetT-epd | pMB1 *epd* sgRNA-pMB1，Spec^r^ | This study |
| pTargetT-mgsA | pMB1 *mgsA* sgRNA-pMB1，Spec^r^ | This study |
| pTargetT-glpD | pMB1 *glpD* sgRNA-pMB1，Spec^r^ | This study |
| pTargetT-gldA | pMB1 *gldA* sgRNA-pMB1，Spec^r^ | This study |
| pFN-Cas9-K | repA101(Ts) kan Pcas-cas9 ParaB-Red lacIq Ptrc-sgRNA-pMB1 pSC10 replication, temperature sensitive replication origin, Para BAD-drivenI-SceI gene, red recombinase expression plasmid, lac-inducible expression; Kan^r^ | This study |

**References**

1. Leang K, Takada G, Fukai Y, Morimoto K, Granström TB, Izumori K. Novel reactions of L-rhamnose isomerase from Pseudomonas stutzeri and its relation with D-xylose isomerase via substrate specificity. Biochimica et biophysica acta. 2004;1674(1):68-77.

2. Lu X, Liu Y, Yang Y, Wang S, Wang Q, Wang X, et al. Constructing a synthetic pathway for acetyl-coenzyme A from one-carbon through enzyme design. Nature communications. 2019;10(1):1378.

3. Peterson PA, Berggård I. Isolation and properties of a human retinol-transporting protein. The Journal of biological chemistry. 1971;246(1):25-33.
